# Role of autophagy in tumorigenesis and drug resistance: molecular mechanisms and therapeutic targets

**DOI:** 10.1186/s43556-026-00430-7

**Published:** 2026-03-12

**Authors:** Jiamin Zhu, Liting Lv, Yuqian Yan, Simin Wang, Xiangdong Lu, Xiaoting Ma, Xia Sun, Ya Qin, Hongshuai Wu, Guiping Yu, Qiong Wang, Xiao Liang

**Affiliations:** 1 Department of Oncology, Jiangyin Clinical College of Xuzhou Medical University, 163# Shoushan Road, Jiangyin, Jiangsu China; 2 Department of Oncology, Affiliated Hospital of Nantong University, Nantong, Jiangsu China; 3 Medical Department, Taizhou Fifth People’s Hospital, Taizhou, China; 4 Department of Cardio-Thoracic Surgery, Jiangyin Clinical College of Xuzhou Medical University, 163# Shoushan Road, Jiangyin, Jiangsu China; 5 Department of Central Laboratory, Jiangyin Clinical College of Xuzhou Medical University, Jiangyin, China

**Keywords:** Autophagy, Drug resistance, Tumorigenesis, Therapeutic target

## Abstract

Autophagy represents a conserved lysosome-dependent catabolic mechanism that safeguards cellular energetic homeostasis and supports adaptive metabolic remodeling under diverse stress conditions. In cancer, autophagy displays a highly context-dependent “double-edged sword” behavior. During the early stages of tumorigenesis, autophagy can suppress malignant transformation by preserving genomic stability, restraining chronic inflammation, and limiting the acquisition of malignant stemness, thereby helping preserve cellular integrity in early tumorigenesis. However, as tumors progress, autophagy can be reprogrammed into an adaptive survival mechanism that supplies tumor growth, metastatic dissemination, and resistance to multiple therapeutic modalities in response to hypoxia, nutrient deprivation, and therapeutic stress. Within the framework of tumor evolution, this review systematically integrates the molecular mechanisms and regulatory networks underlying different forms of autophagy, including canonical, non-canonical, and selective forms. We explore how autophagy intersects with metabolic reprogramming, immune signaling, DNA damage responses, and regulated cell death, and discuss its involvement in tumor progression, microenvironment remodeling, metastasis, and therapy resistance, with relevance to interactions between tumor cells and the surrounding microenvironment. We also summarize recent developments in autophagy-targeted approaches, including chloroquine derivatives, emerging small-molecule inhibitors, and natural compounds, and consider the challenges that remain for clinical translation, especially those related to context-dependent effects and therapeutic application. Collectively, this review provides an updated understanding of autophagy in tumor evolution and informs future mechanistic and therapeutic investigations.

## Introduction

Autophagy, the intricate process responsible for degrading damaged or aging organelles within the lysosome, is regarded as a fundamental regulator of cellular homeostasis [1]. Under physiological conditions, autophagy contributes to key biological processes, including metabolism, development, aging, and the cellular stress response. However, disruptions in autophagy have also been increasingly associated with the pathogenesis of various diseases, such as neurodegenerative disorders, infectious diseases, and metabolic conditions like diabetes [2–5].

In the field of tumor biology, autophagy has attracted particular attention. Genetic and functional studies in both mice and humans have shown that, during the earliest stages of tumorigenesis, autophagy can exert tumor-suppressive effects by preserving genomic integrity, restraining chronic inflammation, and limiting the emergence of malignant stem-like cells. However, once tumors are established, autophagy is often reprogrammed into a pro-survival program that enables cancer cells to withstand multiple adverse stresses, including metabolic stress, detachment from the extracellular matrix, and cytotoxic therapies. This switch constitutes the core of the “double-edged sword” nature of autophagy in cancer [6].

Given the high complexity of autophagy in cancer, elucidating its precise molecular mechanisms and exploring autophagy in depth as a potential therapeutic target are of great importance for both basic and translational research. In this review, we provide an integrated overview of the regulatory framework of autophagy and examine its diverse functions in tumor initiation, progression, and metastatic spread, while discussing current challenges and therapeutic prospects of autophagy-targeted strategies.

## Molecular mechanisms of autophagy

### Core autophagy machinery and signaling pathways

Autophagy is typically triggered by a myriad of intracellular and extracellular stimuli, encompassing stress conditions, fluctuations in energy or nutrient levels, among others. The orchestration of autophagy unfolds with the inception of autophagosomes and double-membrane vesicles that encapsulate diverse cellular constituents. These autophagosomes subsequently merge with lysosomes, resulting in autolysosome formation.

The intricate autophagy pathway is governed by a constellation of proteins belonging to the autophagy-related genes (ATGs) family, with more than 30 identified members. Commencing the cascade, the uncoordinated 51-like kinase (ULK) complex, comprising ULK1, ULK2, ATG13, FIP200, and ATG101, takes center stage [7].

Subsequently, the vacuolar protein sorting 34 (VPS34) complex, recognized as a class III phosphatidylinositol 3-kinase (PI3KC3), is phosphorylated and activated, instigating the generation of phosphatidylinositol-3-phosphate on the phagophore. This event recruits double-FYVE-containing protein 1 proteins (DFCP1) and WD-repeat protein interacting with phosphoinositide (WIPI) proteins, contributing to membrane extension [8].

**Fig. 1:**
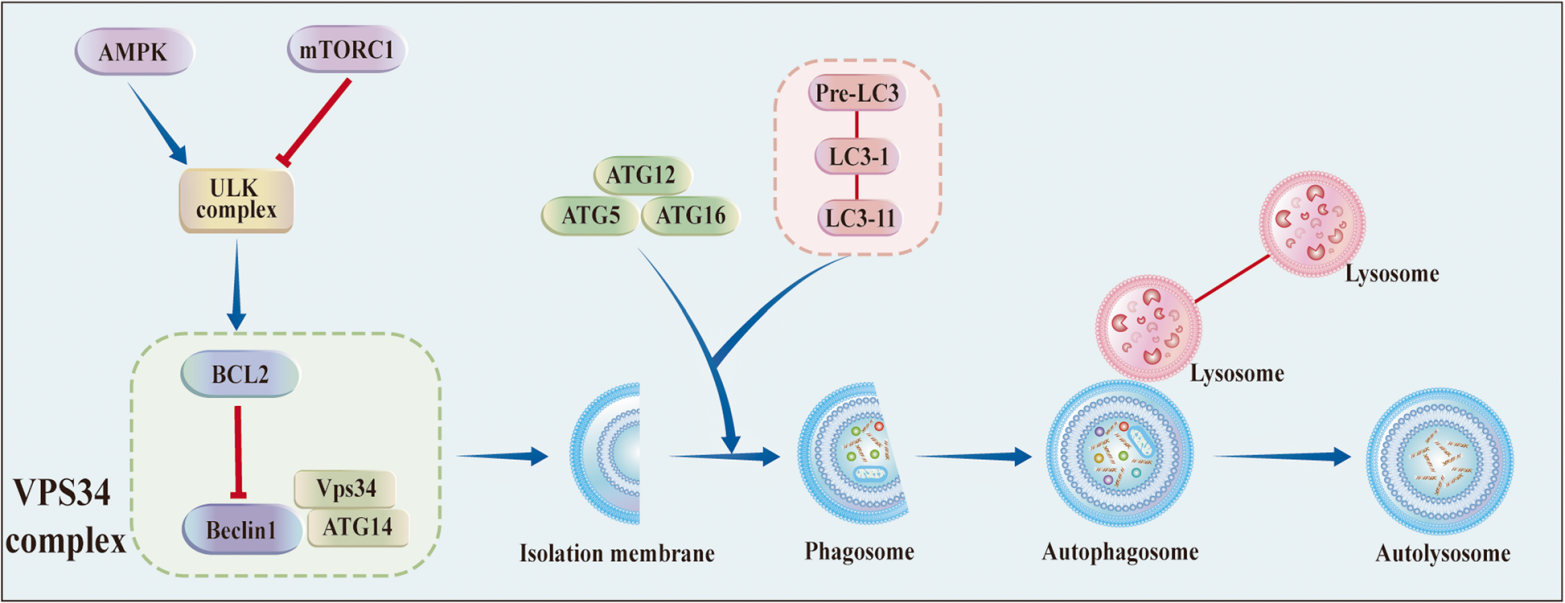
Regulation of autophagy. Autophagy is a multiphasic regulation process involving various ATG (autophagy-related) proteins. The autophagy initiation is physiologically subjected to the stimulatory control of AMP-activated protein kinase (AMPK) or repressive control of the mammalian target of rapamycin complex 1 (mTORC1). ULK complex and VPS34 are involved in the initiation stage of autophagy. Two ubiquitin-like conjugation modules are involved in the expansion of phagophores. After the closure of the phagophore to form an autophagosome, it subsequently fuses with a lysosome to form an autolysosome. Ultimately, this process leads to the autophagic degradation of the substrate

Two ubiquitin-like conjugation systems, the ATG5-ATG12 complex and the ATG8/LC3 system, drive membrane elongation, which interacts with the VPS34 complex to initiate autophagosome formation [9]. Adding a layer of selectivity, autophagy cargo receptors (ACRs) selectively bind to cargo, promoting the specificity of autophagic processes [10]. Upon closure of the phagophore, autophagosomes recruit lysosomal fusion proteins, culminating in the formation of autolysosomes. Here, substrates undergo degradation and subsequent recycling, completing the autophagic cycle. The different steps comprising the autophagy pathway are schematically depicted in Fig. 1.

The regulation of autophagy is controlled by multiple signaling pathways, among which the mTORC1-AMPK axis is considered the central regulatory hub. Under nutrient- and growth factor-replete conditions, mTORC1 is activated, resulting in the phosphorylation and inhibition of the ULK1 complex, thereby blocking the initiation of autophagy [11]. In contrast, during energy deprivation or metabolic stress, AMPK is activated; it can directly phosphorylate and activate ULK1 while simultaneously inhibiting mTORC1, collectively promoting the induction of autophagy. These two kinases function in an antagonistic yet coordinated manner to maintain cellular energy and metabolic homeostasis [12]. In tumors, the PI3K-AKT cascade represents another key pathway involved in autophagy regulation. Upon activation, AKT inhibits the TSC1/TSC2 complex, thereby enhancing RHEB-GTP-mediated activation of mTORC1 and indirectly suppressing autophagy [13]. Additionally, the p53 pathway modulates autophagy in a dual fashion, influenced by its location and activation status. On the one hand, autophagy helps restrain excessive p53 activation by maintaining metabolic stability and clearing reactive oxygen species (ROS) and damaged organelles, thus supporting cell survival [14]. On the other hand, under stress conditions, p53 can transcriptionally activate autophagy-related genes such as DRAM, ULK1, and ATG7 to induce autophagy, thereby accelerating the restoration of cellular homeostasis and the execution of its tumor-suppressive functions [15, 16].

### Non-canonical and selective autophagy

Autophagy is not restricted to canonical autophagy as a single form. In addition to the canonical pathway, multiple non-canonical and selective autophagy routes have been identified in recent years, providing a more refined level of regulation for cellular stress responses and the maintenance of homeostasis.

#### Non-canonical autophagy

Unlike canonical autophagy, which is well-defined by key proteins like Beclin-1 and the Atg gene family, non-canonical autophagy pathways involve alternative mechanisms that can bypass certain steps in the classical autophagic process.

LC3-associated phagocytosis (LAP) is an important form of non-canonical autophagy that utilizes the LC3 conjugation machinery common to canonical autophagy but differs in its induction and specific mechanisms. LAP integrates phagocytosis and LC3 recruitment, driving LAPosome fusion with lysosomes to eliminate pathogens. LC3-associated endocytosis (LANDO) is activated by the recognition of substrates via TREM2 and TLR receptors, followed by clathrin-mediated endocytosis to form endosomes, with LC3 being recruited to LANDOsomes. Other forms of non-canonical autophagy include LANDO and LC3-dependent extracellular vesicle loading and secretion (LDELS). Furthermore, non-canonical autophagy pathways can selectively remove specific intracellular components without relying on the classical autophagosome formation. Additionally, certain receptor proteins, such as OPTN, NBR1, p62/SQSTM1, and NDP52, bind to target substrates and initiate autophagic degradation through various mechanisms. This process also falls under non-canonical autophagy. During the degradation of these substrates, these receptors do not entirely rely on classical autophagosomes and lysosomes; instead, they directly regulate the fusion of target components with lysosomes or facilitate their degradation through alternative pathways.

In summary, non-canonical autophagy represents a flexible and diverse autophagic pathway that, compared to canonical autophagy, plays a critical role under specific conditions and has unique functions in cellular clearance of damaged components and maintenance of cellular homeostasis.

#### Selective autophagy

Based on the specificity of the substrates being degraded, autophagy can also be categorized into non-selective and selective types. Autophagy is classified into selective and non-selective types according to substrate degradation. In selective autophagy, damaged or excess organelles like peroxisomes, mitochondria, lysosomes, ribosomes, nuclei, proteasomes, endoplasmic reticulum, and lipid droplets are specifically degraded and recycled. Representative forms include mitophagy, lipophagy and endoplasmic reticulum (ER)-phagy.

Mitophagy is an important selective autophagic process that removes damaged mitochondria, which can maintain cellular homeostasis and prevent the accumulation of harmful ROS [17]. PINK1 (PTEN-induced kinase 1) and Parkin are the crucial regulatory factors in this process [18–20]. As reported, PINK1-mediated mitophagy promotes the metabolic shift from glycolysis to oxidative phosphorylation (OXPHOS) in drug-tolerant persister (DTP) cells, thereby impelling mitochondrial function and supporting tumor cell survival, while Parkin deficiency-induced mitochondrial dysfunction reduces OXPHOS, increases ROS production, and enhances glycolysis, potentially contributing to the Warburg effect and tumorigenesis [21]. Moreover, several key metabolic signals, such as AMPK [22], protein kinase A (PKA) [23], and the mechanistic target of rapamycin mTOR [24], have been identified as important regulators of mitophagy. The above pathways connect cellular energy status with mitochondrial turnover, ensuring that cells are able to adapt to metabolic changes and stress conditions.

Lipid droplets are intracellular structures that store lipids. Lipophagy is initiated when autophagy receptors interact with lipid droplets, triggering the formation of autophagosomes [25]. These autophagosomes fuse with lysosomes, leading to the degradation of the lipids within the lipid droplets and the release of energy for cellular use. The autophagy receptors involved in lipophagy include LC3, GABARAP, and other autophagy-related proteins [26]. In cancer, lipophagy supports tumor cell survival under metabolic stress by ensuring the proper turnover of lipids [27]. Moreover, lipid-driven autophagy has been shown to impact pivotal cellular processes such as apoptosis, metabolic reprogramming, and immune evasion, making it an essential mechanism for cancer progression and metastasis [28].

The ER plays a vital role in protein synthesis and folding. When the ER is subjected to excessive protein-folding stress or damage, ER-selective autophagy (ER-phagy) is activated to clear abnormal or damaged ER components, thereby alleviating ER stress and restoring cellular homeostasis [29]. ER-phagy relies on specific receptors that bind to the Atg8/LC3/GABARAP family of proteins in autophagosomes, guiding damaged ER components into the autophagic pathway [30]. FAM134B, the first identified ER-phagy receptor, promotes ER fragmentation and clearance through its interaction with LC3/GABARAP. Other receptors include RTN3L, CCPG1, and others [31]. ER-phagy is a key cytoprotective mechanism that plays a dual role under different pathological conditions, promoting cancer cell survival while potentially inducing cancer cell death under certain circumstances.

### Crosstalk between autophagy and other forms of cell death

Autophagy, as a key pathway for cellular self-degradation and recycling, does not operate in isolation but engages in extensive crosstalk with multiple forms of regulated cell death, including apoptosis, ferroptosis, and necroptosis [32].

Autophagy and apoptosis are not mutually exclusive processes. In different cellular contexts, these processes often function in a complementary or antagonistic manner. Mitophagy can reduce mitochondrial permeability and prevent the release of pro-apoptotic factors and cytochrome c into the cytoplasm, thereby suppressing apoptotic cell death [33]. Beclin1, as an essential protein required for the initiation of autophagy, can also interact with anti-apoptotic members of the BCL2 family to regulate the balance between autophagy and apoptosis. In certain cases, the Beclin1-BCL2 interaction can promote autophagy and inhibit apoptosis. Conversely, when autophagy is excessive, Beclin1 is cleaved by caspases, thereby promoting the progression of apoptosis. Apoptosis can likewise regulate autophagy [34]. Popli et al. found that apoptosis can suppress autophagy by activating caspase-3, which reduces the intracellular level of Beclin1 [35].

The relationship between autophagy and ferroptosis also exhibits a dual nature. Mou et al. demonstrated that moderate autophagy suppresses ferroptosis through multiple protective mechanisms [36]. First, autophagy maintains iron homeostasis by preventing excessive ferritin degradation and limiting the release of labile iron, thereby reducing Fenton reaction-driven ROS generation. Second, autophagy activates the p62-Keap1-NRF2 signaling pathway, upregulating antioxidant enzymes such as GPX4 and HO-1 to alleviate lipid peroxidation. In addition, activation of the PI3K-AKT-mTOR and Hippo-YAP/TAZ pathways preserves GPX4 activity and promotes glutathione synthesis, collectively cooperating to inhibit ferroptotic cell death [37]. However, other studies have revealed that excessive activation of selective autophagy promotes iron accumulation and lipid peroxidation, thereby triggering ferroptosis. Several subtypes of autophagy are involved in this process, including NCOA4-mediated ferritinophagy [38], HSP90-regulated chaperone-mediated autophagy [39], RAB7A-driven lipophagy [40], and SQSTM1-associated clockophagy [41]. These selective autophagic processes respectively degrade ferritin, GPX4, and lipid droplets, leading to increased intracellular iron and free fatty acid levels, enhanced lipid peroxidation, and ultimately the induction of ferroptotic cell death. Moreover, several studies have shown that excessive ferroptosis can suppress autophagy, thereby attenuating its dual regulatory role in tumor progression [42].

Necroptosis is a form of regulated necrotic cell death mediated by the RIPK1-RIPK3-MLKL signaling pathway [43]. By weakening the expression of PUMA, mitophagy suppresses mitochondrial outer membrane permeabilisation (MOMP), thereby preventing the release of apoptosis-inducing factor (AIF) and restraining the execution of apoptosis [44, 45]. TRAIL, a canonical death ligand, is capable of activating both apoptotic and necroptotic cascades. When autophagy dampens TRAIL-induced apoptosis, the death signal is diverted towards the RIPK1-RIPK3-MLKL axis, consequently promoting necroptosis [46]. Thus, autophagy acts as a molecular rheostat that fine-tunes the balance between apoptosis and necroptosis by reshaping mitochondrial integrity and redirecting death-ligand signaling.

Autophagy, apoptosis, ferroptosis, and necroptosis are interconnected through complex signaling networks, collectively determining cellular fate and the outcomes of cancer therapy.

### Epigenetic, transcriptional, and post-transcriptional regulation of autophagy

Autophagy regulation occurs through a complex signaling network. Increasing evidence indicates that autophagy is also finely regulated by multiple layers of control, including epigenetic, transcriptional, and post-transcriptional mechanisms.

At the transcriptional level, the expression of autophagy-related genes is primarily governed by two major transcriptional hubs: the MiT/TFE family and the FOXO family. The MiT/TFE family comprises TFEB, TFE3, MITF, and TFEC [47], with TFEB being widely acknowledged as the principal regulator of autophagy and lysosomal biogenesis. Under conditions of nutrient deprivation, mTORC1 inhibition, or lysosomal stress, TFEB translocates from the cytoplasm to the nucleus and binds to CLEAR elements in the promoters of its target genes, thereby transcriptionally activating a broad set of autophagy- and lysosome-related genes [48]. The FoxO family of transcription factors constitutes another key upstream hub in the autophagy regulatory network. Once FoxO is translocated from the cytoplasm into the nucleus, it binds to the promoters of multiple autophagy-related genes and drives their transcriptional activation, thereby regulating several steps of the autophagic process, including initiation, vesicle nucleation, membrane elongation, and autophagosome-lysosome fusion [49]. For example, AMPK-mediated phosphorylation of FoxO3 promotes its nuclear accumulation and stability, thereby enhancing its transcriptional control over autophagy-related genes.

In addition to transcriptional regulation, post-transcriptional regulation also plays a vital role in autophagy. Post-transcriptional regulation of autophagy refers to the mechanisms that control the expression of autophagy-related genes after the transcription of their mRNA. These regulatory processes influence the stability, splicing, translation, and degradation of mRNA molecules, ultimately determining the levels of protein expression and thus modulating autophagic activity within the cell. Non-coding RNAs, including miRNAs and lncRNAs, do not encode proteins but regulate gene stability or translation by interacting with the mRNA of specific autophagy genes. miRNAs can suppress the expression of autophagy genes by binding to their 3’UTRs, while certain lncRNAs interact with chromatin-modifying factors to either promote or inhibit the transcription of autophagy genes [50]. Recent research has revealed that, in addition to miRNAs and lncRNAs, other factors such as RNA-binding proteins, alternative splicing, mRNA stability, and translation regulation collectively govern the autophagic process during post-transcriptional regulation. This complex network ensures the fine-tuning of autophagy in response to cellular signals and environmental stresses.

Epigenetic mechanisms also participate in the regulation of autophagy. For instance, histone acetylation regulates the transcription of autophagy-related genes through modifications to the chromatin structure [51]. DNA methylation usually occurs at the CpG islands in the promoter regions of genes, where it suppresses transcription. The methylation level of the promoter regions of autophagy-related genes, such as Beclin1 and ATG5, determines whether these genes are easily activated [52]. Higher methylation levels may inhibit the expression of autophagy genes, thereby affecting the cell’s autophagic capacity. Each of these factors adds to the intricate nature of epigenetic regulation of autophagy.

In summary, autophagy is not governed by a single pathway, but is finely regulated by a multilayered network composed of transcriptional, post-transcriptional, epigenetic, and multiple upstream signaling mechanisms. These regulatory layers collectively define the intensity, timing, and specificity of the autophagic response in a spatiotemporal manner, enabling cells to precisely adapt to metabolic changes and environmental stress. Conversely, disruption at any of these levels may contribute to the development and progression of various diseases, while also providing multiple potential entry points for autophagy-targeted therapeutic interventions.

### Autophagy and immune signaling pathways

Autophagy is closely interconnected with the immune system and is of great significance in cancer biology. By selectively degrading signaling proteins and "danger signals" in the cytoplasm, autophagy prevents the immune system from overreacting to cellular stress responses. However, cancer cells can also exploit this protective mechanism to evade anti-tumor immune therapies. Autophagy is closely interconnected with immune signaling networks, including NF-κB and STING (stimulator of interferon genes) pathways.

NF-κB is a key regulator of immune responses, inflammation, and cell survival. In acute inflammation, NF-κB helps combat infections, whereas chronic activation of NF-κB can promote tumorigenesis [53]. Previous studies have shown that activation of NF-κB can induce autophagy by enhancing the expression of key autophagic genes, including ATG5, BAG3-HspB8 complex, Beclin1, and LC3. However, NF-κB can also inhibit autophagy by upregulating autophagy suppressors such as Bcl-2 family members, A20, or the PTEN/mTOR signaling pathway [54]. Conversely, autophagy can also regulate NF-κB signaling by degrading components of the IKK complex and NF-κB-inducing kinase (NIK), thereby modulating the NF-κB pathway [55].

The STING pathway is central to the innate immune response to cytosolic DNA. Upon activation, STING triggers the production of IFNs and activates other immune pathways to protect the body from infections and cancer [56]. Studies have shown that autophagy is interconnected with the STING signaling pathway. The activation of STING can enhance the recruitment of autophagic machinery to clear viral or bacterial pathogens. Conversely, autophagy also can function as a negative feedback loop by clearing excessive STING and the activated components of its downstream signaling pathways, such as IFN-β and other pro-inflammatory cytokines, thereby preventing the overactivation of the STING pathway and avoiding detrimental inflammatory responses [57]. Autophagy and the STING pathway collaboratively regulate the immune response, ensuring a rapid and coordinated defense against foreign pathogens.

The interaction between autophagy and immune signaling pathways plays a crucial role in regulating immune responses, with autophagy acting as both a modulator of inflammation and an enhancer of immune activation.

## Autophagy in tumor initiation

Autophagy is generally considered a tumor-suppressive mechanism during the early stages of carcinogenesis. Evidence from genetically engineered mouse models (GEMMs) strongly supports this notion [58–60], and the high-frequency mutations of multiple ATGs observed in human malignancies provide indirect genetic evidence that an intact autophagic program is essential for restraining tumor initiation [59–62]. Based on current evidence, this section will examine the tumor-inhibiting effects of autophagy in tumor initiation, focusing on three interrelated aspects: the preservation of genomic stability, the suppression of chronic inflammation, and the regulation of cellular stemness.

### Autophagy and genomic stability

As outlined above, the maintenance of genomic stability represents a major mechanism by which autophagy exerts tumor-suppressive effects during the earliest stages of carcinogenesis. Through the removal of damaged mitochondria and toxic protein aggregates, autophagy maintains intracellular redox balance and supports genomic stability by curbing excessive ROS [63]. Multiple autophagy-related genes have been identified as key regulators that maintain genomic stability at multiple levels [64]. The loss of the BECN1 gene in ovarian cancer can exacerbate genomic instability to promote tumor initiation [65]. In mice deficient for ATG5 and ATG7, we also observed the development of benign liver tumors, which are associated with genomic damage responses [66]. In a study of autophagy mutant strains of Saccharomyces cerevisiae, deletion of Atg11 was also found to result in impaired mitotic stability of minichromosomes [67]. LC3B, via its RNA recognition motif, directly binds to R-loops at DNA damage sites in transcriptionally active regions, thereby facilitating transcription-associated homologous recombination repair (TA-HRR) and stabilizing BRCA1 mRNA [68]. P62 accumulation leads to enhanced ROS levels, which in turn aggravate genomic instability and promote tumorigenesis [69]. In a study involving 12,427 prostate cancer tissue samples, strong positive p62 expression was also found to be significantly associated with all detected genomic deletions, suggesting that p62 accumulation may be related to genomic instability [70]. Namely, elevated p62 levels may inhibit DNA damage-induced histone ubiquitination, disrupting chromatin stability [71]. Autophagy-related proteins such as ATG7, Beclin1, Parkin, and FIP200 have also been reported to suppress tumor initiation by regulating the cell cycle through a non-autophagic pathway [64, 72].

### Autophagy and inflammation

Beyond its role in maintaining genomic stability, autophagy also constrains chronic inflammation, which constitutes an additional critical barrier to tumor initiation during the early stages of carcinogenesis. Early studies revealed that many core autophagy genes are associated with inflammatory diseases, among which ATG16L1 has been identified as a risk allele for Crohn’s disease. Subsequently, an increasing number of studies have clearly demonstrated that autophagy plays a crucial role in the regulation of inflammation [73, 74]. Chronic inflammation is widely recognized as a key factor in the development of numerous cancers, including those driven by persistent infections such as hepatitis B and C virus-associated liver cancer and Helicobacter pylori-associated gastric cancer, as well as by toxin exposure such as alcohol-related liver cancer and smoking-related lung cancer [75]. Autophagy can attenuate cytokine production during hyperinflammation and infection [76]. Diverse elements of air pollution can promote lung carcinogenesis by inhibiting autophagy, thereby triggering local inflammation [77]. Autophagy can also attenuate the activation of the NLRP3 inflammasome in Helicobacter pylori-associated gastritis. Atg7-deficient adipose tissue exhibits an imbalance of oxylipins and reduced circulating IL-10 levels, thereby exacerbating intestinal inflammation [78]. Elevated hepatocyte autophagy can facilitate IL-1β/TNF-induced necrosis by impairing energy homeostasis, causing lysosomal permeabilization and enhancing inflammation via exosome release of damage-associated molecular patterns [79]. The loss of p62 significantly alleviates liver injury caused by autophagy deficiency [80]. Autophagy has also been reported to inhibit HBV replication [81].

### Autophagy in the maintenance of stemness and cancer stem cells (CSCs)

Beyond its functions in maintaining genomic stability and restraining inflammation, autophagy also plays an important role in the regulation of cellular stemness and CSCs [82]. This function, which regulates stemness, plays a key role in tumor suppression during the initial stages of tumorigenesis. It prevents normal cells from becoming CSCs by maintaining genomic integrity and cellular homeostasis. Once the malignant transformation occurs and normal cells acquire CSC properties, autophagy becomes essential for maintaining their stem-like characteristics [83]. In addition to sustaining stemness, autophagy also exerts a critical role in restraining the differentiation of CSCs. One mechanism involves the selective degradation of differentiation-inducing proteins, such as the cyclin-dependent kinase inhibitors p16 and p21, which would otherwise drive cell-cycle exit and terminal differentiation. By removing these factors, autophagy helps CSCs maintain an undifferentiated phenotype. Moreover, autophagy intersects with multiple signaling pathways, including WNT, Notch and TGF-β, to coordinately regulate CSC self-renewal, differentiation and survival [84].

Although abundant evidence supports a tumor-suppressive role of autophagy during the early stages of tumor initiation, several studies have also highlighted important limitations and controversies. Heterozygous deletion of BECN1 in mice has been associated with increased tumor susceptibility, and genomic alterations of BECN1 have likewise been observed in human breast cancer; however, subsequent studies have challenged these findings, suggesting that BECN1 loss may be co-linear with alterations at the neighboring BRCA1 locus, and thus whether BECN1 itself qualifies as a bona fide tumor suppressor remains to be fully clarified [85]. In Ras-driven tumors, Beclin has been shown to promote MDM2-mediated degradation of p53 [86], and in mouse models of chemically induced hepatocarcinogenesis, increased autophagic activity accompanied by excessive inflammatory responses has been observed [87]. Taken together, these findings suggest that, in the early phases of tumorigenesis, autophagy primarily functions as an important barrier by preserving genomic stability, restraining pro-inflammatory signaling, and modulating tumor cell stemness, yet the oncogenic consequences of autophagy loss or aberrant activation must be interpreted with caution. It is crucial to distinguish whether these effects arise from a global loss of autophagic function or from context-dependent dysregulation of specific autophagy pathways or components.

## Autophagy in tumor progression and promotion

**Fig. 2:**
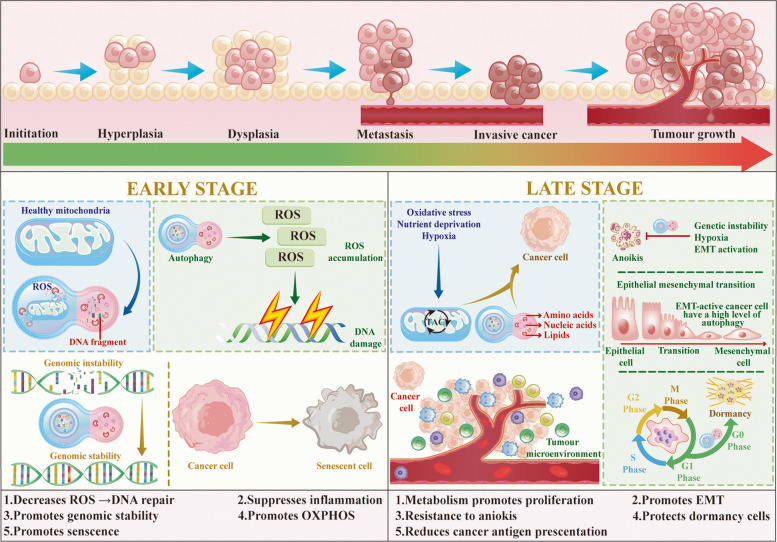
The dynamic roles of autophagy in tumor initiation and progression. Autophagy plays a dynamic role across different stages of tumor progression, from initiation to invasive cancer, and its contribution to cancer therapy. In the early stages of cancer, autophagy acts as a protective mechanism by reducing ROS, promoting genomic stability, and enhancing OXPHOS, thereby preventing DNA damage and facilitating cellular senescence. As the tumor progresses, oxidative stress, nutrient deprivation, and hypoxia within the tumor microenvironment, can drive autophagy for supporting tumor cell survival, proliferation, and epithelial-mesenchymal transition (EMT), while also aiding resistance to anoikis. In the late stages, autophagy enhances tumor cell metabolic reprogramming, promotes EMT, and protects dormant cells from therapy-induced damage

During the early stages of tumorigenesis, autophagy primarily acts as an overarching tumor-suppressive barrier by maintaining genomic stability, suppressing chronic inflammation, and limiting the acquisition of malignant stemness; however, as tumors progress, autophagy gradually shifts to a driver of cancer evolution, participating in multiple aspects of tumor biology, including metabolic reprogramming, tumor microenvironment remodeling, and metastatic dissemination [88]. Figure 2 provides a systematic overview of the dynamic roles of autophagy across the continuum of tumor progression from initiation to invasive disease and delineates its contributions to cancer therapy. The following sections provide a more detailed discussion of how autophagy operates in these processes.

### Autophagy and cancer metabolism

Autophagy, as a central pathway for intracellular material and energy redistribution, has a close, bidirectional regulatory relationship with metabolic reprogramming in tumors: on the one hand, autophagy provides essential substrates for tumor cells under metabolic stress; on the other hand, different metabolic pathways in turn shape the level and pattern of autophagy, thereby jointly driving tumor evolution [89].

The interplay between autophagy and glycolysis can determine the progression of cancer. In colorectal cancer, METTL16 enhances glycolysis and promotes tumor progression by regulating the SOGA1/PDK4 axis [90]. LncRNA LINRIS was found to stabilize IGF2BP2 by blocking its K139 ubiquitination-mediated degradation depending on the autophagy-lysosome pathway, thereby maintaining MYC-driven aerobic glycolysis to promote cancer progression [91]. Cystatin B enhances autophagic flux in pancreatic cancer by sustaining the proteolytic activity of cathepsin B, thereby fueling glycolysis to support tumorigenesis [92]. In breast cancer, chaperone-mediated autophagy (CMA) enhances angiogenesis by upregulating hexokinase 2 (HK2)-dependent aerobic glycolysis, thereby increasing lactate production and vascular endothelial growth factor A (VEGFA) expression, which in turn drives tumor progression and is associated with poor prognosis [93].

Beyond glucose metabolism, cancer cells also exhibit alterations in lipid metabolism. Autophagy-mediated regulation of lipid metabolism sustains tumor cell survival and proliferation under metabolic stress and promotes cancer cell acclimatization to the tumor microenvironment. In colorectal cancer, Timosaponin AIII (TA-III) has been shown to induce lipophagy via Rab7-mediated pathways, enhancing lipid droplet degradation and promoting tumor progression [94]. As a plant-derived bioactive compound, Rottlerin (Rott) induces lipid modification of LC3 and activates autophagy and apoptosis in prostate cancer stem cells through the PI3K/Akt/mTOR signaling pathway [95]. Yang et al. demonstrated that CD36, as a fatty acid receptor, regulated the interplay between lipid metabolism and autophagy, driving metabolic reprogramming and tumor progression in cancer cells [96]. In pancreatic cancer, FABP5 modulates lipophagy by promoting lipid droplet deposition and lipid metabolism, thereby supporting tumor cell growth and metastasis [97]. However, the role of autophagy in regulating lipid metabolism has a typical “double-edged sword” nature and displays marked context dependence. Excessive activation of autophagy can lead to excessive degradation of lipids, suppress lipid synthesis, and result in energy depletion in the cell, potentially promoting tumor cell death [98–100]. This phenomenon can be observed in ATG14-mediated lipophagy in HeLa cells [101].

Although cancer cells mainly rely on aerobic glycolysis to meet their energy demands, mitochondrial OXPHOS still makes an important contribution to ATP production [102]. Mitophagy regulates mitochondrial function to establish a dynamic balance between these two processes [21]. In the early stages of tumorigenesis, mitophagy helps to suppress tumor formation by removing dysfunctional mitochondria and improving the TME [103]. In breast cancer, urolithin A has been reported to promote mitophagy by activating TFEB, which can reduce harmful inflammatory factors such as IL-6 and TNF-α, and inhibit tumor progression. As tumors advance, mitophagy may transition from a tumor-suppressive to a tumor-promoting role. This shift occurs because mitophagy enables cancer cells to adapt to the TME, which is typically characterized by hypoxia, nutrient deprivation, and oxidative stress [104]. As is demonstrated that in conditions of glucose deprivation, MANF translocates to the mitochondria, where it interacts with PRKN and alleviates oxidation. This action restores PRKN’s E3 ligase activity, promoting mitophagy and enhancing fatty acid oxidation (FAO), thereby supporting the survival of breast cancer cells [105]. In bladder cancer, DARS2 promotes tumor progression via regulating PINK1-mediated mitophagy, thereby inhibiting cellular senescence and enhancing the proliferation of bladder cancer cells [106].

### Autophagy and tumor microenvironment (TME)

**Fig. 3:**
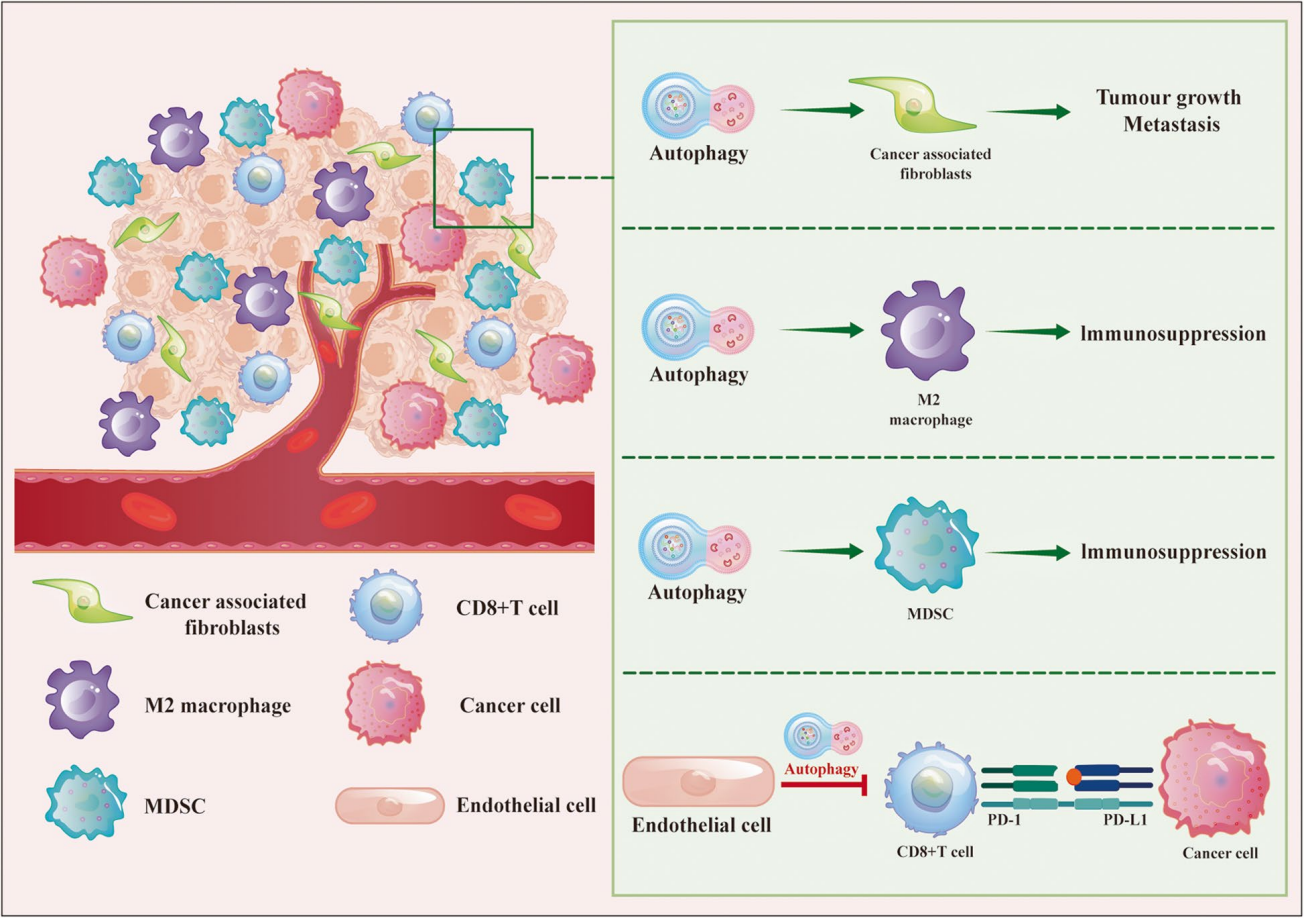
The multifaceted role of autophagy in tumor progression and tumor microenvironments. In cancer-associated fibroblasts, autophagy promotes tumor progression by enhancing the invasive and proliferative capabilities of the tumor. In M2 macrophages and MDSCs, autophagy facilitates immunosuppression, allowing tumors to evade immune surveillance. Additionally, autophagy in endothelial cells impacts the interaction between tumor cells and CD8 + T cells, further promoting immune escape through the PD-1/PD-L1 axis. This figure highlights the complex interplay between autophagy and immune cells within the TME and its potential as a therapeutic target to overcome treatment resistance and enhance anti-tumor immunity

Autophagy, by finely tuning cellular metabolism, remodels the energy supply within cancer cells and endows them with metabolic adaptability, while also reshaping the TME by altering the activation state and function of cancer associated fibroblasts (CAFs), immune cells and endothelial cells, thereby fostering a milieu that favors tumor growth [107, 108]. Figure 3 summarizes the multilayered, context-dependent roles of autophagy within these distinct cellular compartments in driving malignant tumor phenotypes.

As a specialized population of stromal cells, CAFs are crucial in promoting tumor progression within the TME [109]. Previous studies have demonstrated that autophagic activity in the TME is an essential mechanism for the activation of fibroblasts and the formation of CAFs [110]. Narita et al. found that CAFs supply key metabolites, including lactate, ketone bodies, and fatty acids, to support the energy demands of neighboring tumor cells [111]. CAFs influence tumor behavior by secreting cytokines, growth factors, and remodeling the extracellular matrix (ECM) [112]. In lung cancer, inhibition of CAF autophagy reduces tumor cell EMT and metastatic potential via NF-κB signaling. In a bladder cancer TME model, increased autophagy in CAFs enhances tumor cell proliferation, invasion, and metabolic reprogramming [113]. In pancreatic cancer, researchers have found that pancreatic stellate cells differentiate into CAFs through autophagy, regulating the secretion of alanine and providing critical metabolic support to tumor cells [114]. Furthermore, in tumor radiotherapy, CAFs can promote autophagy in tumor cells by secreting factors such as IGF1/2 and CXCL12, aiding in the repair of radiation-induced damage and contributing to tumor relapse [115].

Autophagy is also involved in the regulation of immune cell [116]. Inhibition of autophagy has been demonstrated to enhance immune surveillance by upregulating the surface expression of major histocompatibility complex class I (MHC-I) molecules on cancer cells, thereby facilitating T-cell-mediated cytotoxicity [117]. Previous investigations have also illustrated that inhibiting CDKL3 expression reduces autophagy induction, enhances M1 macrophage polarization, strengthens the immune response, and increases the infiltration of immune-activating cells in esophageal cancer [118]. TRAF2 has been confirmed to promote M2 macrophage polarization by inhibiting autophagy [119]. Caprin-1 can initiate autophagy through its interactions with ULK1 and STK38, thereby increasing the infiltration of tumor-associated macrophages (TAMs) [120]. Autophagy also plays a key regulatory role in myeloid-derived suppressor cells (MDSCs). HMGB1-induced autophagy promotes the polarization of MDSCs and enhances their immunosuppressive function, thereby facilitating immune escape [121], whereas Xia et al. reported that silencing LDHA enhances autophagy, inhibits MDSC recruitment, and strengthens anti-tumor immune responses [122]. Jelle et al. found that autophagy in Tumor endothelial cells (TECs) restricts immune cell infiltration, including CD8 + T cells, by reducing the expression of immune-attracting chemokines and adhesion molecules in melanoma models. The loss of autophagy in TECs enhances T-cell infiltration and amplifies anti-tumor immune responses [123].

Autophagy in endothelial cells regulates angiogenesis. Inhibition of angiogenesis in human dermal microvascular endothelial cells is observed upon treatment with chloroquine (CQ) or 3-MA [124]. Conversely, in tumor cell studies under hypoxic conditions, inoculation of B16F10 melanoma cells into wild-type or Beclin-1 knockdown mice reveals increased angiogenesis, with larger tumors and more lung metastases observed in Beclin-1 knockdown mice [125]. TECs are the main structural components of tumor blood vessels and perform a crucial function in the tumor stroma. Autophagy is reported to regulate the survival and function of endothelial cells, influencing the extent of tumor vascularization [126]. In breast cancer, autophagy promotes the differentiation of breast cancer stem cells into endothelial-like cells, contributing to tumor vascularization and progression [127].

### Autophagy and tumor metastasis

Tumor metastasis remains the leading cause of cancer-related mortality. During metastasis, cancer cells must overcome the local anatomical and matrix constraints of the primary tumor, acquire sufficient motility to detach from the extracellular matrix via integrin-dependent cytoskeletal remodeling, and ultimately colonize distant organs to establish secondary lesions [128, 129]. In this highly dynamic metastatic process, autophagy is extensively engaged and coordinately regulates key events such as tumor cell invasion, EMT, anoikis resistance and tumor dormancy, thereby shaping the overall course of tumor metastasis. In the following sections, we systematically discuss the role of autophagy in tumor metastasis with a focus on these critical steps.

Tumor invasion is the initial step in the metastatic process, where cancer cells must breach the basement membrane and ECM to enter the bloodstream [130]. Recent studies have increasingly demonstrated that autophagy directly contributes to tumor cell invasion during migration and metastasis [131–133]. Autophagy not only enhances tumor cell motility by promoting cytoskeletal remodeling but also regulates the activity of matrix metalloproteinases (MMPs), facilitating the degradation of the ECM and augmenting the invasive capacity of tumor cells [134, 135]. In addition, autophagy is reported to heighten RAS-driven tumor invasion and metastasis via upregulating the secretion of pro-invasive factors such as IL-6, MMP2, and WNT5A [136]. Chen et al. found that YAP promoted the migration of TNBC cells by transcriptionally activating genes such as ANKRD1, which are associated with cellular invasiveness [137].

During the process by which tumors invade surrounding tissues from the primary site and enter the bloodstream, EMT serves as a key transitional program [138]. In melanoma, MCOLN1-mediated autophagy inhibition disrupts EMT and suppresses cancer metastasis by modulating the ROS-driven TP53/p53 pathway [139]. In other tumor models, such as gastric [140], colorectal [141], and breast cancer [142], autophagy has been found to accelerate tumor metastasis by enhancing EMT. Autophagy can also facilitate tumor progression by fulfilling the energy requirements of highly invasive and metastatic cancer cells [143]. However, the role of autophagy in EMT is dualistic. It has been reported that autophagy can suppress EMT by degrading p62 and the transcription factor TWIST1 [144]. Similarly, research has demonstrated that CDH6 (a member of the Atg8 subfamily) interacts with GABARAP (a TGF-β target gene) in the EMT process to inhibit the degradation phase of autophagy, thereby promoting cytoskeletal reorganization and further facilitating EMT and tumor cell metastasis in thyroid cancer [145]. Furthermore, autophagy and EMT can achieve a dynamic balance in response to changes in the TME. In the model of pancreatic ductal adenocarcinoma, EMT serves as a prerequisite for invasion. However, autophagy is essential for maintaining organelle stability and metabolic homeostasis. Premature cell death, in the absence of autophagy, impedes the metastatic process [146].

Anoikis is a form of programmed cell death, which can be triggered by detachment from the ECM. Normal epithelial and endothelial cells undergo apoptosis upon detachment from the matrix, a protective mechanism to prevent ectopic growth [147]. However, during metastasis, tumor cells must acquire anoikis resistance to survive the bloodstream or lymphatic circulation and facilitate their spread [148]. Gao et al. found that GDF15 enhanced the survival of gastric cancer cells after detachment by activating protective autophagy, which increased anoikis resistance and facilitated tumor metastasis [149]. In breast cancer, tumor cells can downregulate mTOR to initiate autophagy, which makes for the survival of tumor cells upon ECM detachment by promoting nutrient recycling, organelle turnover, and mitochondrial maintenance [150]. This process enhances anoikis resistance and contributes to metastasis.

Tumor dormancy refers to the process by which tumor cells enter a prolonged state of quiescence or low proliferation [151]. Dormant cells can include CSCs, late-stage metastatic dormant cells, or treatment-induced surviving cells in a quiescent state [152]. Various studies have reported that malignant cells increase autophagic activity during the dormant phase [153]. This dormant state can persist for months to years and is one of the key contributors to tumor recurrence and drug resistance [154]. In an experiment, researchers transplanted breast cancer cells into microenvironments that either restricted or allowed dormancy. Inhibition of autophagy leads to the accumulation of damaged mitochondria and ROS, subsequently inducing apoptosis in dormant cells. However, this effect has minimal impact on proliferating cells, suggesting that autophagy inhibition specifically targets dormant tumor cells, with little effect on proliferating cells [155]. The MAPK pathway has been reported to centrally regulate tumor metastasis [156]. Research across various cancer cell types, including breast, prostate, and melanoma, has elucidated the role of the MAPK pathway in cancer dormancy [157]. In colorectal cancer, downregulation of PLK4 induces autophagy through the MAPK pathway, resulting in cell entry into dormancy [158]. Similarly, ARHI triggers autophagy and tumor cell dormancy via inhibiting the PI3K-AKT signaling pathway in ovarian cancer [159]. However, the role of autophagy in tumor dormancy is also complex and paradoxical. Aqbi et al. found that chemotherapy-induced autophagy can facilitate tumor relapse from dormancy, whereas cell-intrinsic autophagy, by reducing DNA damage and genomic instability, can delay tumor recurrence [160]. Evaluating the autophagic activity of tumors before and after chemotherapy, particularly the levels of cell-intrinsic autophagy, may help predict the dormancy status of tumor cells and the risk of recurrence. With the advancement of molecular technologies, researchers can now utilize techniques such as single-cell RNA sequencing to observe the gene expression differences between dormant and other tumor cells, providing new technical tools for investigating the mechanisms of tumor dormancy [161].

In summary, autophagy is an indispensable regulatory mechanism in tumor metastasis, with an increasing body of research uncovering its multiple critical functions throughout the metastatic process. Targeting specific stages in the metastatic cascade with autophagy inhibitors offers the potential to reduce cancer mortality. Furthermore, the dual role of autophagy in metastasis suggests that interventions should carefully consider tumor type, microenvironment, and therapeutic stage.

## Molecular mechanisms of autophagy in drug resistance

Autophagy, functioning as both a survival and death mechanism, plays a critical role in drug resistance in cancer, positioning itself as a promising therapeutic target. Accumulating evidence suggests that therapy-induced activation of autophagy plays a pivotal role in enabling cancer cells to evade drug-induced cytotoxicity, thereby contributing to the emergence of treatment resistance [162–164]. In specific genetic or metabolic contexts, autophagy may exert tumor-suppressive functions by facilitating the degradation of resistance-associated factors such as mutant p53, YAP, or dysfunctional mitochondria, thereby reversing therapeutic resistance [165–167]. Owing to its dual roles in tumor promotion and suppression, dysregulated autophagy can either potentiate or mitigate drug resistance across diverse cancer contexts. Only through rational and context-specific targeting of autophagy can drug resistance be effectively overcome. However, the multifaceted functions of autophagy in cancer render its role in drug resistance even more intricate [168].

**Fig. 4:**
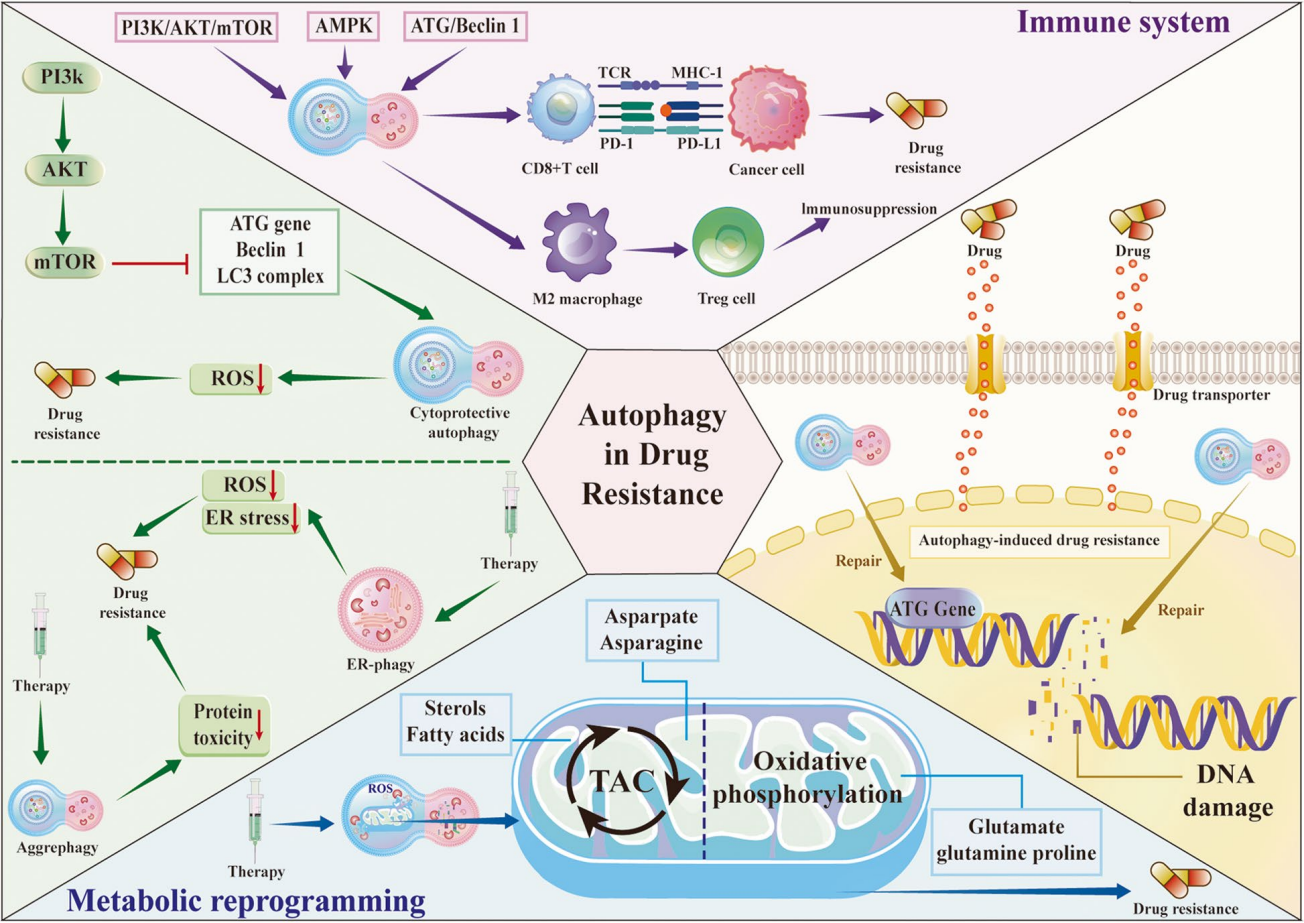
The multifaceted roles of autophagy in cancer drug resistance. Autophagy exerts complex and context-dependent effects in cancer drug resistance through multiple interconnected mechanisms, including metabolic reprogramming, immune modulation, DNA damage repair, and cellular stress adaptation. In the immune system, autophagy regulates the activity of cytotoxic CD8 + T cells, M2 macrophages, and Treg cells, leading to immunosuppression and resistance to immune checkpoint blockade via the PD-1/PD-L1 axis. Key signaling pathways, including PI3K/AKT/mTOR, AMPK, and ATG/Beclin-1 complex, govern autophagy initiation and cytoprotective autophagy that enhances tumor survival under therapeutic stress. In metabolic reprogramming, autophagy contributes to the degradation of misfolded proteins (aggrephagy) and damaged endoplasmic reticulum (ER-phagy) to relieve ER stress and ROS accumulation, thereby promoting drug resistance. Autophagy supports mitochondrial metabolism by sustaining the tricarboxylic acid (TCA) cycle and OXPHOS, supplying metabolites such as aspartate, asparagine, glutamate, and fatty acids essential for tumor adaptation. In the DNA damage response, autophagy facilitates DNA repair and autophagy-induced drug resistance by regulating ATG gene expression and drug transporter activity

This section synthesizes how autophagy shapes therapeutic resistance by buffering cellular stress, reprogramming key signaling pathways, engaging selective autophagy subtypes and drug efflux mechanisms. Table 1 and Fig. 4 summarize the role of autophagy in cancer drug resistance.

**Table 1:** Mechanisms of autophagy in regulating drug resistance

Tumor type	Gene	Function in Autophagy Regulation	Autophagic Proteins	Therapy	Drug	Role of autophagy in resistance of therapy	Reference
NSCLC	COPS3 FOXO3	• COPS3 facilitates FOXO3 nuclear translocation • Enhances the expression of LC3 and RAB7 • Promotes autophagy	LC3 RAB7	Chemotherapy	Cisplatin	Upregulation	[169]
SCLC	GGPS1	• GGPS1 upregulation promotes RAB7A activation • Activates autophagy • Removes detrimental levels of oxidative stress, damaged DNA or misfolded protein induced by chemotherapy	LC3B RAB7A	Chemotherapy	VP16 + DDP	Upregulation	[170]
NSCLC	GMI CD133	• GMI induces CD133 protein degradation via autophagy	LC3 P62 ATG5	Chemotherapy	Pemetrexed	Upregulation	[171]
Osteosarcoma	HSP90AA1	• HSP90AA1 promotes autophagy through the PI3K/Akt/mTOR pathway • Inhibits apoptosis through the JNK/P38 pathway	LC3 P62	Chemotherapy	Doxorubicin Cisplatin Methotrexate	Upregulation	[172]
SCLC	NRBF2	• NRBF2 interacts with P62 to form autophagic P62 bodies • Promotes autophagy	P62	Chemotherapy	Cisplatin VP-16	Upregulation	[173]
Lung Cancer Breast Cancer Liver Cancer	USP24	• USP24 downregulation induces the expression of LC3 • Promotes autophagy • Upregulates E2F1 and enhances ULK1 levels, further stimulating autophagy • Maintains genomic stability	LC3	Chemotherapy/Target therapy	Taxol Gefitinib	Downregulation	[174]
LUAD	LOC85009	• LOC85009 regulates USF1 to downregulates ATG5 expression • Inhibits autophagy	ATG5	Chemotherapy	Docetaxel	Upregulation	[175]
LUAD	SNHG7	• SNHG7 recruits HuR (Human Antigen R) to stabilize ATG5 and ATG12 mRNAs • Promotes autophagy	ATG5 ATG12	Chemotherapy	Docetaxel	Upregulation	[176]
Ovarian Cancer	TXNDC17	• TXNDC17 regulates autophagy by modulating the expression of Beclin 1 • Promotes autophagosome formation and autophagic flux	Beclin 1 LC3 ATG5	Chemotherapy	Paclitaxel	Upregulation	[177]
Glioblastoma Multiforme	PSMC2	• PSMC2 suppresses JNK-mediated autophagic cell death • Reduces autophagy	Beclin 1 LC3 ATG7 P62	Chemotherapy	Temozolomide	Downregulation	[178]
Breast Cancer	CLDN6	• CLDN6 induces protective autophagy via LKB1/AMPK/ULK1 signaling	ULK1 LC3B P62 LKB1	Chemotherapy	Adriamycin Paclitaxel	Upregulation	[179]
Glioblastoma	SOCS5	• SOCS5 promotes autophagy via Bcl-2 regulation • Facilitates the formation of autophagosomes • Protects GBM cells against TMZ-induced cell death	Bcl-2 Beclin 1 LC3 ATG7 p62	Chemotherapy	Temozolomide	Upregulation	[180]
HSCC	RAB3B	• RAB3B upregulates autophagy by amplifying genes through eccDNA • Activates Bcl-2 • Protects tumor cells against chemotherapy-induced cell death	LC3B Beclin1 P62 ATG7	Chemotherapy	Cisplatin	Upregulation	[181]
Osteosarcoma	FOXM1	• FOXM1 enhances autophagy through the HMMR/ATG7 signaling pathway • Removes damaged organelles and proteins	LC3 Beclin1 ATG7,	Chemotherapy	Methotrexate	Upregulation	[182]
Colon Cancer	KLF4	• KLF4 inhibits autophagy by targeting RAB26 • suppresses the formation of autophagosomes and reduces 5-FU resistance	LC3 P62	Chemotherapy	5-Fluorouracil	Upregulation	[183]
B- Cell C- Lymphomas	S100A8	• S100A8 enhances the expression of autophagic proteins like BNIP3, BECN1, and PI3KC3 • Activates autophagy • helps cells survive under stress conditions, including exposure to chemotherapy agents	LC3 P62 Beclin1	Chemotherapy	Adriamycin + Vincristine	Upregulation	[184]
NSCLC	DSTYK	• DSTYK inhibits mTORC1 • Promotes autophagy • Increases sensitize • Decreases the sensitivity of the TNF-α-mediated CD8 + killing	P62 LC3	Immunotherapy	PD-1 inhibitors	Upregulation	[185]
NSCLC	Trim35	• Trim35 inhibits LSD1 activity via ubiquitination • Enhances ERGIC1 transcription • Suppresses autophagy • Promotes CD8 + T cell infiltration and PD-L1 level	LC3 P62 ERGIC1	Immunotherapy	PD-1 inhibitors	Downregulation	[186]
Lung Cancer	PD-L1	• Autophagy inhibition promotes the aggregation of dsDNA • Activates the cGAS-STING pathway • Promotes immune activation and enhances the efficacy of PD-L1 blockade	LC3 P62	Immunotherapy	Radiotherapy combined with PD-L1 inhibitors	Upregulation	[187]
Colorectal Cancer	ATG16L1	• ATG16L1 promotes autophagosome elongation • Suppresses IFN responses and tumor T cell infiltration	LC3 ATG8 P62	Immunotherapy	Atezolizumab	Upregulation	[188]
NSCLC	PPARγ	• PPARγ regulates PD-L1 expression by inducing autophagic degradation of PD-L1 • Reduces immune escape and enhancing T-cell activity • PPARγ facilitates PD-L1 degradation through its interaction with LC3	LC3 ATG7	Immunotherapy	PD-L1 inhibitors	Downregulation	[189]
TNBC Prostate Cancer	Sigma1	• Sigma1 inhibitors induce autophagic degradation of PD-L1 • Reduces immune evasion and promotes T-cell activation	LC3 ATG5 ATG7	Immunotherapy	PD-L1 inhibitors	Downregulation	[190]
Breast Cancer Lung Cancer	CDK5	• CDK5 degrades PD-L1 by mediating chaperone-mediated autophagy • Preservs the immune activity of T cells	LC3 Beclin1 ATG7 P62	Immunotherapy	PD-1 inhibitors	Upregulation	[191]
NSCLC	IL-17A	• IL-17A blocks autophagy via the ROS/Nrf2/p62 pathway • Increasing PD-L1 levels and inhibits PD-L1 degradation • Promotes immune escape	LC3 P62	Immunotherapy	PD-L1 inhibitors	Upregulation	[192]
NSCLC	ULK1	• ULK1 downregulation inhibits autophagy • Induces NK cell-mediated cell killing	ATG5 ATG7 Beclin-1	Immunotherapy	–	Upregulation	[193]
Pancreatic Cancer	TMOD3	• TMOD3 enhances autophagosome-lysosome fusion through F-actin polymerization • Protects cells from the effects of ferroptosis	LC3	Immunotherapy	PD-1 inhibitors	Upregulation	[194]
PDAC	NDRG1	• NDRG1 promotes the stability of MHC-1 by enhancing the autophagic function of ATG9A • Impairs the effective clearance by the immune system • Promotes immune evasion	LC3 P62 ATG9A	Immunotherapy	PD-1 inhibitors CTL4 inhibitors	Upregulation	[195]
NSCLC	OPTN	• OPTN enhances mitophagy • Stabilizes MHC-1 expression, enhancing tumor cell recognition by CD8 + T cells • Promotes immune evasion	LC3 P62	Immunotherapy	PD-L1 inhibitors	Upregulation	[196]
TNBC	ATAD3A	• ATAD3A inhibits PINK1-mediated autophagy • Enhances PD-L1 accumulation	PINK1 LC3 LAMP1	Immunotherapy Chemotherapy	Paclitaxel PD-L1 inhibitors	Downregulation	[197]
Hepatocellular Carcinoma	miR-25	• MiR-25 inhibits the expression of FBXW7, • Activates of autophagy-related pathways • Removes sorafenib-induced damaged organelles	LC3 P62 Beclin1	Targete therapy	Sorafenib	Upregulation	[198]
Hepatocellular Carcinoma	SCAP	• SCAP overexpression inhibits autophagy through AMPK signaling • Reduces the cell death induced by sradenib	LC3-II P62	Target therapy	Soradenib	Downregulation	[199]
NSCLC	ATG16-L1	• ATG16-L1 splicing leads to ATG16-L1 β-isoform • Inhibits autophagic flux, prevents cells from clearing drug-induced damaged	LC3 P62	Target therapy	Gefitinib Dacomitinib	Downregulation	[200]
NSCLC	CD74-ROS1	• CD74-ROS1 induces autophagy via the MEK/ERK pathway • Protects tumor cells from drug-induced damage	LC3 Beclin1 P62	Target therapy	Crizotinib	Upregulation	[201]
NSCLC	AXL	• High expression of AXL enhances autophagy • Degrades drug-induced damaged proteins and dysfunctional mitochondria	LC3 Beclin1 P62	Target therapy	Erlotinib	Upregulation	[202]
NSCLC	ALK FOXO3A	• Lorlatinib inhibits ALK activity, reduces the level of phosphorylated AKT • Promotes autophagy • Forms a protective barrier that counteracts the drug’s cytotoxicity	LC3 P62	Target therapy	Lorlatinib	Upregulation	[203]
Multiple Myeloma	Bim	• Bim interacts with Beclin-1 to inhibit autophagy • Enhances cell death and restore tumor cell sensitivity to bortezomib	LC3 Beclin1	Target therapy	Bortezomib	Upregulation	[204]
CML	KIF23	• KIF23 promotes autophagy by enhancing LC3-II accumulation and p62 expression • regulates Wnt/β-catenin signaling • clears the damage caused by imatinib	LC3-II p62 Beclin1	Target therapy	Imatinib	Upregulation	[205]
Colorectal Cancer	Beclin-1	• Beclin-1 promotes autophagy • Removes drug-induced damaged substances and reduce cell death	LC3-II Beclin-1 P62	Target therapy	Bevacizumab	Upregulation	[206]

*NSCLC* Non-Small Cell Lung Cancer, *SCLC* Small Cell Lung Cancer, *HSCC* Hypopharyngeal Squamous Cell Carcinoma, *PDAC* Pancreatic Ductal Adenocarcinoma, *TNBC* Triple-negative breast cancer, *LUAD* Lung Adenocarcinoma, *CML* Chronic Myeloid Leukemia

### Cytoprotective autophagy and drug resistance mechanisms

Autophagy is a catabolic and evolutionarily conserved process whereby intracellular components and damaged organelles are degraded or recycled via lysosomal activity, thereby preserving cellular homeostasis and metabolic equilibrium [207]. Upon exposure to chemotherapeutic agents, targeted therapies, or radiotherapy, cancer cells frequently upregulate autophagic flux through the activation of core signaling cascades such as AMPK, inhibition of mTOR, and modulation of the PI3K/AKT pathway [208, 209]. Enhanced autophagy promotes the degradation and recycling of damaged organelles and proteins, mitigates the accumulation of ROS, and supports metabolic reprogramming to meet bioenergetic demands [210]. Consequently, anticancer therapies induce genotoxic, oxidative, or metabolic stress in tumor cells, and autophagy acts as a cytoprotective buffer during this process, promoting cancer cell adaptation and survival [211]. When autophagy facilitates resistance to treatment and its inhibition enhances cell death, it is classified as cytoprotective [212].

Qian et al. demonstrated that in hepatocellular carcinoma, internalization of CD147 and lysosomal translocation of its complex with G3BP1 induced cytoprotective autophagy, thereby reducing tumor cell sensitivity to chemotherapy by suppressing the mTOR signaling pathway [213]. Similarly, kinase-dead PRKD1 enhances prostate cancer cell survival by inducing STK11-dependent cytoprotective autophagy and activating the LKB1-AMPK signaling pathway, thereby conferring resistance to therapeutic stress [214]. Studies have shown that lncRNAs such as H19, MALAT1, and NEAT1 can activate autophagy by interacting with EZH2, DNMT1, or miRNA pathways, thereby promoting resistance to chemotherapy, radiotherapy, or targeted therapies. These lncRNAs enhance a cytoprotective autophagic state that favors cancer cell survival by epigenetically silencing or activating key ATGs such as ATG5, ATG7, or Beclin-1 [215–218]. Notably, inhibition of autophagy or disruption of lncRNA-mediated epigenetic regulatory loops has been shown to restore chemosensitivity in various tumor models. Emerging evidence has identified JD-02, a recently characterized HSP90 inhibitor, that overcomes therapy resistance in triple-negative breast cancer by converting cytoprotective autophagy into a pro-death mechanism and reversing EMT-associated survival pathways [219]. In another study of chemoresistant triple-negative breast cancer cells, overexpression of BAG3 was shown to drive cytoprotective autophagy, thereby enhancing anti-apoptotic capacity and enabling sustained cell survival under chemotherapeutic stress. Notably, both pharmacological and genetic inhibition of autophagy or BAG3 expression effectively restored tumor cell sensitivity to chemotherapy [220]. Exemestane (Exe), a potent steroidal aromatase inhibitor, has been demonstrated to promote cytoprotective autophagy in models of acquired resistant breast cancer. Inhibition of autophagy and/or the PI3K pathway in these models restores Exe sensitivity by enhancing apoptosis, disrupting cell cycle progression, and suppressing pro-survival signaling pathways [221].

### Selective autophagy subtypes and drug resistance

Recent advances have revealed that, beyond bulk autophagy, selective autophagy subtypes play pivotal roles in mediating drug resistance in cancer by facilitating the targeted degradation of specific cellular components [222]. This form of autophagy not only helps cells adapt to therapeutic stress but also directly contributes to the survival of tumor cells under various stress conditions induced by chemotherapy, targeted therapy, or radiotherapy [223]. The main selective autophagy subtypes, mitophagy, ER-phagy, and aggrephagy, each serve distinct yet complementary roles in supporting cancer cell resistance to treatment.

The mechanisms underlying mitophagy have been outlined in the preceding sections. Mitochondria are major sites of ROS production, and their dysfunction can lead to increased ROS, which in turn promotes cell death [166]. However, cancer cells frequently exploit mitophagy to mitigate oxidative stress and prevent mitochondrial dysfunction-induced apoptosis, a key mechanism in the development of drug resistance. For example, in response to chemotherapy-induced oxidative damage, tumor cells upregulate mitophagy to selectively remove damaged mitochondria, relieving ROS accumulation and ensuring cellular survival [211]. This process has been reported to be associated with resistance against numerous anticancer agents, including cisplatin, targeted therapies, and radiation [58].

Recent research by Zhang et al. reveals that mitophagy mediated by the LINC01607/miR-892b/p62-Nrf2 pathway promotes lenvatinib resistance in hepatocellular carcinoma by enhancing mitochondrial quality control and maintaining redox homeostasis. Inhibition of this signaling axis effectively restores tumor sensitivity to lenvatinib treatment [224]. Another study has shown that in a variety of malignancies, including hepatocellular carcinoma, lung cancer, and breast cancer, elevated expression of ATAD3A inhibits PINK1-Parkin-dependent mitophagy by preventing the accumulation of PINK1 on the outer mitochondrial membrane. This mechanism enables tumor cells to evade apoptosis triggered by mitochondrial damage and thereby confers resistance to multiple chemotherapeutic agents, such as cisplatin [197]. Limagne et al. recently reported that activation of the KRAS-MEK pathway suppresses OPTN-dependent mitophagy, thereby reducing the efficacy of chemoimmunotherapy in cancer. Inhibition of MEK restores mitophagic flux, triggers mitochondrial DNA-dependent TLR9 signaling, and enhances recruitment of CD8 + T cells, collectively sensitizing tumors to combination chemotherapy and immunotherapy [196]. In multiple myeloma (MM), researchers have found that lomitapide, a cholesterol-lowering drug, promotes mitochondrial dysfunction and enhances cell survival under therapeutic stress by activating DRP1-mediated mitophagy in MM cells, leading to chemotherapy resistance [225]. The precise role and molecular mechanisms of dysregulated mitophagy in tumor initiation, progression, and chemoresistance remain to be fully elucidated and require extensive investigation. Moreover, in vivo studies and prospective clinical trials are warranted to validate the efficacy of drugs or small-molecule inhibitors targeting mitophagy for overcoming drug resistance.

ER homeostasis is fundamental for maintaining cellular proteostasis and facilitating adaptation to diverse stress conditions [226]. In cancer, therapeutic interventions often impose excessive demands on protein folding or provoke the persistent accumulation of misfolded proteins, thereby eliciting chronic ER stress [227, 228]. To counteract these insults, tumor cells orchestrate a repertoire of adaptive responses, among which ER-phagy has recently emerged as a crucial cytoprotective mechanism [229].

ER-phagy is a selective form of autophagy that relies on ER-resident receptors such as FAM134B, SEC62, RTN3, ATL3, and TEX264 to specifically recognize and deliver damaged or excess ER fragments to lysosomes for degradation [230]. Recent investigations have revealed that ER-phagy, by efficiently removing dysfunctional ER segments, significantly alleviates proteotoxic stress and supports tumor cell survival under therapeutic pressure, thus facilitating the development of drug resistance [231].

Moreover, the unfolded protein response (UPR) and ER-phagy are tightly interconnected adaptive mechanisms that collectively safeguard ER proteostasis under stress. For instance, in MYC-driven breast cancer, oncogenic MYC directly activates the IRE1/XBP1 arm of the UPR, which maintains ER proteostasis and enables tumor cells to withstand proteotoxic stress, thereby contributing to chemoresistance [232]. Additionally, in another study on breast cancer, FAM134B/BiP-mediated ER-phagy is activated by hypoxia-induced proteotoxic stress, allowing cancer cells to survive chemotherapeutic challenge, while inhibition of FAM134B enhances drug sensitivity [233]. Taking colorectal cancer as an example, Zhang et al. demonstrated that the ALK inhibitor brigatinib induces ER stress centered on the FAM134B-LC3B axis, promoting tumor cell apoptosis while simultaneously activating ER-phagy [234]. This process removes damaged or expanded ER regions, effectively alleviates excessive ER stress, and limits drug-induced cell death. Notably, both pharmacological inhibition (such as with chloroquine) and genetic suppression (such as FAM134B knockdown) of ER-phagy significantly enhance tumor cell sensitivity to brigatinib and other chemotherapeutic agents.

Aggrephagy, the selective autophagic clearance of aggregated or misfolded proteins, plays a critical but underappreciated role in tumor resistance to therapy. When cancer cells are exposed to treatments that induce protein misfolding or inhibit protein degradation (such as chemotherapy or proteasome inhibitors), protein aggregates accumulate and trigger proteotoxic stress [235, 236]. Aggrephagy is induced to degrade these toxic protein aggregates, thereby alleviating stress, preventing apoptotic signaling, and maintaining cellular homeostasis [237].

Recent research has revealed that CCT2-mediated aggrephagy constitutes a pivotal survival mechanism for cancer cells under therapeutic stress. Investigators have demonstrated that CCT2, through its non-canonical VLIR domain, interacts with ATG8 family members to specifically recognize and clear insoluble protein aggregates [238]. This process enables tumor cells to alleviate proteotoxic stress induced by chemotherapy or targeted agents, thereby supporting cellular adaptation and survival in hostile treatment environments. Young et al. reported that in lung cancer cell lines exhibiting resistance to Taxol or gefitinib, pharmacological inhibition of USP24 with USP24-i-101 enhanced autophagic flux, reduced protein aggregation, and restored drug sensitivity [174]. Moreover, transcriptomic analyses in breast cancer have revealed that aggrephagy-related gene signatures such as TUBA1C, TUBA3D, TUBA3E, TUBB1, and VIM can stratify patients into distinct prognostic and drug-response subgroups. In high-risk tumors, upregulation of these genes promotes the efficient clearance of cytotoxic protein aggregates generated during treatment, alleviates proteotoxic stress, suppresses apoptosis, and sustains cellular viability under cytotoxic challenge. This adaptive mechanism enables cancer cells to tolerate otherwise lethal doses of chemotherapy, ultimately driving the emergence of drug resistance [239]. Although mechanistic studies on the relationship between aggrephagy and drug resistance remain limited, existing evidence suggests that selective autophagic clearance of protein aggregates may offer a promising strategy to overcome therapeutic resistance in cancer. Further targeted research will be essential to advance this field.

### Autophagy and specific resistance mechanisms

Autophagy can support tumor cell survival and confer therapeutic resistance through multiple independent yet interconnected mechanisms. Extensive studies have demonstrated that autophagy in tumors often assumes a cytoprotective role, helping cancer cells resist genotoxic, oxidative, and metabolic stress [228]. Consequently, targeting autophagy has emerged as a promising combination therapeutic strategy. In the future, developing modulators that selectively inhibit autophagy-while accounting for context dependence and safety-will be critical to overcoming treatment resistance and enhancing the efficacy of chemotherapy, targeted therapy, and radiotherapy.

Firstly, DNA repair and autophagy represent distinct yet complementary biological processes that are essential for maintaining cellular survival and genomic stability. Impairment of autophagy disrupts efficient DNA damage repair and sensitizes cells to apoptosis in response to genotoxic insults [240, 241]. Evidence indicates that autophagy sustains cellular energy homeostasis, provides nucleotides, and regulates the levels of DNA repair proteins essential for accurate DNA replication and repair [242]. Accumulating data suggest that autophagy enhances resistance to genotoxic therapies such as chemotherapy and radiotherapy by facilitating DNA damage repair [243, 244]. Research on glioblastoma has revealed that temozolomide exposure upregulates MEX3A, which accelerates MSH2 mRNA degradation and suppresses mismatch repair activity. This impairment not only disrupts autophagy and apoptosis activation upon DNA damage, but also allows tumor cells to evade genotoxic stress and develop acquired chemoresistance [245]. In pancreatic cancer, Xiao et al. demonstrated that upregulation of NLRP4 confers resistance to the PARP inhibitor olaparib by simultaneously promoting the DNA damage response and ROS-induced autophagy. Mechanistically, NLRP4 augments DNA repair capacity and elevates mitochondrial ROS production, which in turn activates cytoprotective autophagy pathways, collectively enabling tumor cells to withstand therapeutic stress [246].

Moreover, autophagy critically modulates antigen processing and presentation, thereby attenuating the efficacy of anticancer immunotherapies. It has been well established that autophagy facilitates the degradation of tumor antigens and MHC-I molecules within cancer cells, leading to reduced antigenicity and enabling tumor cells to escape immune surveillance, which ultimately results in resistance to immunotherapeutic interventions. For example, in pancreatic cancer, autophagy selectively targets and degrades MHC-I molecules through NBR1-mediated recognition, resulting in a decreased abundance of MHC-I on the cell surface [117]. This process suppresses tumor immunogenicity, impairs antigen presentation to cytotoxic T lymphocytes, and ultimately attenuates anti-tumor immune responses, thereby promoting resistance to immune checkpoint blockade therapies. Furthermore, selective autophagy also regulates the turnover of immune checkpoint proteins, such as PD-L1. For instance, Research has shown that impairment of autophagy leads to the accumulation of PD-L1 on tumor cells, which enhances the interaction between PD-1 and T cells, thereby inhibiting T cell activation and promoting resistance to immunotherapy [247]. In addition, mechanistic studies in prostate cancer have revealed that increased extracellular matrix stiffness activates the integrin β1/FAK/YAP-USP8/NBR1 signaling axis, which accelerates selective autophagy-mediated degradation of MHC-I and increases the stability of PD-L1, thereby fostering an immunosuppressive and contributing to immunotherapy resistance [248]. Autophagy is further implicated in modulating dendritic cell cross-presentation and antigen processing, as it affects phagosome-lysosome fusion and the degradation of antigens [249]. Notably, oncogenic pathways-including PI3K/AKT/mTOR, AMPK, and ATG5/7-Beclin-1 signaling cascades-are frequently dysregulated in tumors, leading to enhanced autophagic flux, particularly under immunotherapeutic pressure [107, 250]. This dysregulation not only diminishes tumor antigenicity and hampers the function of cytotoxic lymphocytes, but also fosters the recruitment of regulatory T cells and myeloid-derived suppressor cells, thereby strengthening the immunosuppressive environment and contributing to immune resistance.

Thirdly, Autophagy-driven metabolic reprogramming constitutes a central adaptive mechanism that underlies therapeutic resistance in cancer [251]. In the context of antitumor therapy, cancer cells experience metabolic stress due to nutrient deprivation, hypoxia, and therapy-induced cytotoxicity [252]. To overcome these challenges, tumor cells activate autophagy to degrade and recycle intracellular macromolecules, thus providing an alternative source of metabolic substrates such as amino acids, fatty acids, and nucleotides [253]. This process sustains ATP production, redox balance, and biosynthetic pathways, enabling cancer cells to endure and adapt to harsh microenvironmental and therapeutic conditions [254]. Recent evidence demonstrates that autophagy-driven metabolic plasticity also plays a pivotal role in mediating resistance to a wide spectrum of anticancer therapies [255, 256]. In response to treatment-induced stress, tumor cells activate autophagy to recycle intracellular components, thereby sustaining essential metabolic pathways including glycolysis, glutaminolysis, and fatty acid oxidation [257]. Particularly, in a study on pancreatic ductal adenocarcinoma (PDAC), researchers have found that autophagy maintains glutamine metabolism, thereby supporting tumor cell survival under metabolic stress. Pharmacological inhibition of autophagy sensitizes PDAC cells to gemcitabine, highlighting the critical role of autophagy in drug resistance [258]. Moreover, in various cancer types, enhanced autophagic flux has been shown to clear damaged mitochondria and limit excessive ROS accumulation, thus preventing apoptosis and sustaining cell survival during therapeutic pressure [259]. Additionally, oncogenic signaling pathways such as PI3K/AKT/mTOR and AMPK not only regulate autophagy but also orchestrate metabolic adaptation in the context of therapeutic stress. Dysregulation of these pathways results in increased autophagic flux and metabolic plasticity, which together facilitate tumor cell adaptation, survival, and the development of drug resistance. For instance, in estrogen receptor-positive breast cancer, resistance to endocrine therapy is driven by enhanced fatty acid oxidation and increased autophagy, both under the control of AKT/AMPK signaling [260]. Xiao et al. found that chemotherapy-induced upregulation of HSP90AA1 promotes autophagy-mediated chemoresistance in osteosarcoma via the PI3K/Akt/mTOR pathway, and that autophagy inhibition restores drug sensitivity [172]. Notably, inhibition of either fatty acid oxidation or autophagy restores drug sensitivity in resistant cells [261, 262]. Collectively, these findings underscore that autophagy-mediated metabolic reprogramming has emerged as a universal strategy underpinning tumor drug resistance. Accordingly, combined targeting of autophagy and metabolic pathways represents a promising approach to overcome therapeutic resistance across a spectrum of malignancies.

### Autophagy and efflux mechanisms in drug resistance

Drug efflux is one of the major mechanisms by which tumors acquire drug resistance [263]. Recent studies have shown that autophagy plays a critical regulatory role in drug efflux through various mechanisms, including providing energy, modulating transporter turnover, and offering alternative “bypass” efflux pathways [264]. Autophagy and drug efflux interact in a complex, dual-edged manner in the context of tumor drug resistance.

ATP-binding cassette (ABC) transporters, such as P-glycoprotein (P-gp), breast cancer resistance protein (BCRP), and multidrug resistance-associated proteins (MRPs), actively pump chemotherapeutic agents out of tumor cells, thereby reducing intracellular drug accumulation and undermining therapeutic efficacy [265]. During the adaptive phase of resistance development, autophagy and drug efflux transporters collaborate to promote cell survival, thereby driving resistance. Giddings et al. discovered that in drug-resistant cells, the ABCB1 and ABCG2 transporters preferentially utilize ATP derived from mitochondrial OXPHOS, rather than solely relying on the cytosolic ATP pool [266]. This finding suggests that the function of these transporters is largely dependent on mitochondrial integrity, which is primarily maintained by autophagy, especially mitophagy. In this context, autophagy supports the sustained activity of drug efflux pumps by ensuring ATP supply, thereby promoting drug resistance through the maintenance of mitochondrial function under chemotherapy-induced stress. Thus, autophagy not only plays a role in maintaining cellular energy homeostasis but also contributes to resistance by supporting the function of efflux pumps. However, this defense balance is fragile. When autophagy is excessively activated or specifically enhanced, ABC transporters may be directed to lysosomal degradation, thereby weakening drug efflux function. Certain natural compounds, such as curcumin derivatives, can regulate autophagic flux, induce P-gp ubiquitination, and recruit it via p62/SQSTM1 to autolysosomes, effectively reversing resistance by “dismantling” the efflux pump [267].

In addition, secretory autophagy has emerged as a significant and increasingly recognized mechanism of drug resistance, distinct from the ABC transporter-mediated efflux [268]. When ABC pump function is compromised or pharmacologically inhibited, cancer cells can activate an alternative efflux pathway through secretory autophagy. In this process, drugs or other exogenous molecules are encapsulated in autophagosomes, which do not fuse with lysosomes for degradation [269]. Instead, these vesicles fuse with the plasma membrane, releasing their contents into the extracellular space via exosomes. This allows cancer cells to expel drugs and maintain resistance, even when ABC pumps are inhibited or not upregulated [270].

Understanding the interaction mechanisms between autophagy, drug efflux, and metabolic reprogramming provides new strategies for overcoming resistance, particularly by targeting autophagy and metabolic regulatory pathways, which could effectively enhance the efficacy of anticancer drugs.

## Targeting autophagy in cancer therapy

In view of the critical roles of autophagy in tumor initiation, progression and therapeutic resistance, pharmacological modulation of autophagy has increasingly been recognized as an important therapeutic strategy in oncology. Multiple classes of small molecules capable of altering autophagic activity have now been identified. Building on robust preclinical data and the long-standing clinical use of CQ and its derivative hydroxychloroquine (HCQ) in malaria and rheumatoid arthritis, numerous clinical trials have been launched to repurpose these agents as inhibitors of tumor autophagy [271, 272]. In parallel, new generations of small molecules that more selectively target key components of the autophagy-lysosome machinery, as well as a variety of natural products with autophagy-modulating properties, have shown encouraging antitumor activity in preclinical models. In the following sections, we provide a systematic overview of the current landscape of autophagy-targeted strategies, with a particular focus on CQ/HCQ, emerging synthetic agents and natural product-derived autophagy modulators, and we discuss the major obstacles that still limit the successful translation of autophagy modulation into routine cancer therapy.

### Clinical application of chloroquine derivatives as autophagy inhibitors

CQ and HCQ have emerged as the most intensively studied autophagy inhibitors, with investigations spanning chemotherapy, targeted therapy, and immunotherapy [273–275]. The earliest clinical trial of autophagy inhibitors was a small study in glioblastoma multiforme, in which chloroquine was administered as an adjuvant therapy to the standard regimen, including surgery, radiotherapy, and chemotherapy [276]. Although this study used a randomized controlled design, the sample size was small (only 9 of 18 postoperative patients received an additional 150 mg of oral chloroquine), and all participants were enrolled after surgery, which imposes substantial limitations on the conclusions. Notably, chloroquine-related adverse events were limited to seizures and were generally manageable, providing preliminary support for its safety. Another clinical investigation showed that erlotinib combined with 1,000 mg HCQ was safe and tolerable, but its clinical efficacy was still limited [277]. In a separate single-arm study, the amalgamation of hydroxychloroquine with chemotherapy among patients with metastatic NSCLC showcased a potentially enhanced objective response rate (ORR) in those harboring KRAS mutations [278]. Conversely, a study focusing on KRAS lung cancer illustrated the limited antitumor efficacy of the combination therapy involving a MEK inhibitor and HCQ, leading to premature trial termination. Moreover, a concerning 55.6% of patients experienced grade 3 adverse events (AEs) [279]. In advanced breast cancer, a phase I trial of HCQ plus low-dose palbociclib and letrozole demonstrated acceptable safety, with some patients achieving partial responses or stable disease; importantly, clinical response was associated with dynamic changes in autophagy markers such as p62 and LAMP1, suggesting that autophagy inhibition may potentiate endocrine and CDK4/6 inhibitor therapy [280]. To date, trials with reported results are listed in Table 2.

**Table 2:** Clinical trials target autophagy

Tumor type	Autophagy inhibitor	Combination	Clinical Trial phase	Results	National Clinical Trial Number
NSCLC	HCQ	Erlotinib	Phase I	No dose-limiting toxicities DLTs were observed. The MTD of HCQ with erlotinib was 1000 mg daily. The recommended phase II dose for HCQ was 1000 mg when combined with erlotinib 150 mg daily. HCQ did not affect erlotinib pharmacokinetics	NCT01026844
Advanced solid tumors Melanoma	HCQ	Temozolomide	Phase I	No DLTs observed. MTD not reached. Recommended phase II dose of HCQ 600 mg twice daily with dose-intense TMZ. 14% PR and 27% SD in melanoma patients	NCT00714181
Advanced solid tumors Melanoma	HCQ	Temsirolimus (mTOR inhibitor)	Phase I	No DLTs observed. MTD for HCQ not reached. The recommended phase II dose for HCQ was 600 mg twice daily with Temsirolimus 25 mg weekly. Median progression-free survival: 3.5 months for melanoma Promising antitumor activity, especially in melanoma	NCT00909831
Advanced solid tumors	HCQ	Vorinostat (VOR, HDAC inhibitor)	Phase I	DLTs: Fatigue and gastrointestinal toxicities (Grade 3 fatigue, anemia). MTD was determined as 600 mg HCQ with 400 mg VOR daily. The recommended phase II dose for HCQ was 600 mg twice daily with 400 mg VOR daily. One patient with RCC achieved a durable PR lasting over 50 cycles SD observed in CRC and STS patients. No significant impact on the PK of VOR	NCT01023737
Hepatocellular Carcinoma	GNS561	-	Phase I	No DLTs observed. MTD for HCQ not reached. The recommended phase II dose for HCQ was 300 mg GNS561BID No CR or PR was observed; 5 patients (25%) achieved SD. 15 patients (75%) had PD	NCT03316222
Glioblastoma Multiforme	HCQ	Radiation Therapy (RT) and TMZ	Phase I/II	Phase I: DLTs: 800 HCQ daily. MTD of HCQ was 600 mg daily. Phase II: Median survival of 15.6 months. Significant increases in autophagic vacuoles and LC3-II in peripheral blood mononuclear cells were observed, correlating with HCQ exposure. Autophagy inhibition was not consistently achieved with 600 mg HCQ. No significant improvement in OS was observed with HCQ addition	NCT00486603
Metastatic pancreatic adenocarcinoma	HCQ	-	Phase II	DLTs: not determined MTD: not determined Median progression-free survival (PFS): 46.5 days. OS: 69.0 days. Inconsistent autophagy inhibition was observed in patients’ peripheral lymphocytes when assessed by LC3-II levels	NCT01273805
Advanced metastatic BRAF V600E mutant cutaneous melanoma (Stage IV)	HCQ	Dabrafenib (BRAF inhibitor) Trametinib (MEK inhibitor)	Phase I/II	DLTs: not determined MTD: not determined No clinically significant ocular events or ocular inflammation observed	NCT02257424
Renal cell carcinoma	HCQ	Everolimus (mTOR inhibitor)	Phase I/II	No DLTs observed MTD of HCQ was 600 mg twice daily Disease control (SD + PD) in 22 of 33 patients (67%) 6-month PFS in 45% of patients Median PFS: 6.3 months	NCT01510119
Pancreatic adenocarcinoma	HCQ	Gemcitabine	Phase I/II	No DLT observed MTD of HCQ was 1200 mg/day 61% of patients had a decrease in CA 19–9 77% R0 resection rate Median OS: 34.8 months Disease-free survival improvement with LC3-II response	NCT01978184
Metastatic castration-resistant prostate cancer	Pantoprazole	Docetaxel + Prednisone	Phase II	No DLT observed Recommended Doses: pantoprazole 240 mg once every 21 days PSA response rate: 52% (11/21) Radiographic partial response rate: 31% (4/13) Median OS: 15.7 months Median PFS: 5.3 months	NCT01748500
NSCLC	HCQ	Carboplatin + paclitaxel	Phase I/II	No DLT observed Recommended Doses: HCQ 200 mg BID ORR:33% Median PFS was 6.4 months	NCT00728845
Resectable solid tumors	HCQ	Surgical rection	Phase I	DLTs: not determined MTD: not determined Recommended Doses: HCQ 200 mg BID Apoptosis observed in patients with elevated Par-4 67% of patients showed a two-fold increase in Par-4 No clinically significant ocular events observed	NCT02232243
Metastatic colorectal cancer	HCQ	VOR	Phase II	No DLT observed Recommended Doses: HCQ was 600 mg/day Median PFS with Vorinostat + HCQ: 1.9 months vs Regorafenib: 4.35 months (*P* = 0.032) Median OS for Vorinostat + HCQ: 6.77 months vs Regorafenib: 7.23 months (*P* = 0.90)	NCT02316340

Data are collected from US National Library of Medicine ClinicalTrials.gov

*DLTs* dose-limiting toxicities, *MTD* maximum tolerated dose, *PR* response rate, *SD* stable disease, *PK* pharmacokinetics, *PD* progressive disease, *PFS* progression-free survival

In summary, HCQ and CQ clinical trials have faced several limitations, including inconsistent autophagy inhibition, limited efficacy in advanced and chemoresistant cancers, and poor clinical benefit in terms of survival outcomes. Adverse events and challenges in dosing optimization have further complicated results. Variability in patient responses suggests a need for individualized treatment strategies. Future trials should focus on refining dosing, improving patient stratification, and developing more specific autophagy inhibitors to overcome these challenges and improve clinical outcomes.

### Next-generation autophagy inhibitors: from drug development to clinical translation

As the limitations of traditional autophagy inhibitors in terms of selectivity and safety have become increasingly apparent, a new generation of autophagy inhibitors with higher specificity and more favorable pharmacokinetic properties has emerged and is gradually advancing toward clinical translation. Table 3 summarizes all ongoing Phase I and II clinical trials. Most of these trials are still in the early stages (Phase I/II) with small sample sizes. These studies aim to explore more specific and potent autophagy inhibitors, such as GNS561 (Ezurpimtrostat) and VPS34 inhibitors, which focus on blocking the early stages of autophagy. Additionally, these studies attempt to combine autophagy inhibitors with established therapies, such as immune checkpoint inhibitors and chemotherapy, to enhance therapeutic efficacy and overcome drug resistance.

**Table 3:** Ongoing clinical trials target autophagy

Tumor type	Autophagy inhibitor	Treatment	Clinical Trial phase	National Clinical Trial number
Metastatic melanoma	HCQ	HCQ/nivolumab HCQ/nivolumab/Ipilimumab	Phase I/II	NCT01550367
Hepatocellular carcinoma	GNS561	Atezolizumab/Bevacizumab	Phase II	NCT05448677
Advanced melanoma	HCQ	Nivolumab/Ipilimumab	Phase I/II	NCT04464759
Resectable localized prostate cancer	HCQ	Surgical rection	Phase II	NCT06408298
Breast cancer	HCQ	Avelumab/Palbociclib	phase II	NCT04841148
Resectable pancreatic adenocarcinoma	HCQ	mFOLFIRINOX	Phase I/II	NCT04911816
Advanced chemorefractory solid tumors	HCQ	CPI-613 (devimistat) + 5-fluorouracil (5-FU)/gemcitabine	Phase II	NCT05733000
Advanced KRAS mutated cholangiocarcinoma	GNS561	Trametinib	Phase I/II	NCT05874414

Data are collected from US National Library of Medicine ClinicalTrials.gov

GNS561 is an orally available lysosomotropic small molecule and a clinical-stage inhibitor of palmitoyl-protein thioesterase 1 (PPT1). By selectively targeting PPT1, GNS561 perturbs lysosomal acidity and the activity of multiple hydrolases, induces lysosomal dysfunction, thereby blocking late stages of autophagy and triggering lysosome-dependent cell death. This agent exhibits pronounced liver tropism and has displayed robust antitumor activity in multiple in vitro and in vivo models of hepatic malignancies, including hepatocellular carcinoma and intrahepatic cholangiocarcinoma [281]. In a phase I dose-escalation study in patients with advanced hepatocellular carcinoma and cholangiocarcinoma, oral GNS561 at 200 mg BID demonstrated an overall acceptable safety profile, liver exposure comparable to efficacious levels observed in preclinical models, and disease stabilization in a subset of patients, thereby laying the groundwork for subsequent phase II evaluation [282].

In addition, VPS34, playing a central role in the initiation of autophagy, has emerged as an important novel target for autophagy-directed therapy. At the initiation stage of autophagy, VPS34 is the key lipid kinase responsible for generating PI3P and driving phagophore nucleation, and is therefore regarded as an important upstream drug target for autophagy modulation. In recent years, multiple highly selective ATP-competitive VPS34 small-molecule inhibitors, such as PIK-III, SAR405, VPS34-IN1, and 36–077, have been developed; by blocking PI3P production and autophagosome formation, they effectively suppress both basal and stress-induced autophagy [283]. A series of studies have shown that these inhibitors not only directly restrain tumor cell proliferation and invasion, but also markedly enhance the sensitivity to tyrosine kinase inhibitors, cisplatin, and immune checkpoint inhibitors, thereby overcoming autophagy-mediated drug resistance [284]. In addition, indirect inhibitors such as Spautin-1, which promote degradation of the VPS34 complex, and dual-target agents like MPT0L145 that simultaneously inhibit VPS34 and FGFR, further broaden the spectrum of VPS34-based strategies for therapeutic autophagy modulation [285]. However, VPS34-targeted agents remain largely at the preclinical stage, and their selectivity, safety profile, and bona fide on-target activity in vivo still need substantial optimization before they can realistically underpin large-scale, rigorously designed clinical development programs.

### Autophagy-targeting natural products

In addition to HCQ, CQ and GNS561, numerous natural products have amassed substantial preclinical evidence in vitro and in vivo, modulating the autophagy-lysosome axis in various cancers [286, 287]. These natural substances possess the ability to either induce or inhibit autophagy, thereby enhancing their potential in overcoming drug resistance. For instance, in lung cancer, Xie-Bai-San (XBS), a traditional Chinese medicine formulation containing key components like Kukoamine B, Mulberroside A, and Liquiritin, has shown promise in overcoming EGFR-TKI resistance by impeding autophagy initiation through the inhibition of Beclin-1 [288]. Similarly, Feiyanning (FYN) has been found to sensitize lung cancer cells to cisplatin by inhibiting autophagy through a reduction in autophagosome formation [289]. Berberine, a natural compound, influences chemosensitivity by modulating both protective and lethal forms of autophagy. Administration of berberine leads to upregulation of MAPK1/ERK2-MAPK3/ERK1, activates autophagy, and enhances the sensitivity of glioblastoma to temozolomide [290]. Propofol (2,6-diisopropylphenol) is an intravenous anesthetic widely used for the induction and maintenance of anesthesia. Studies have shown that propofol can enhance the sensitivity of lung cancer cells to cisplatin chemotherapy by inhibiting autophagy and increasing the expression levels of MIR744-5p and MIR615-3p [291]. Additionally, propofol administration can enhance cisplatin sensitivity in gastric cancer by downregulating the expression of MALAT1 and upregulating MIR30E, a known inhibitor of ATG5 translation [292].

Additional herbal compounds have demonstrated the ability to induce autophagy. Pomiferin, a natural product derived from Osage Orange, has been identified as an autophagy activator. It triggers autophagic cell death in cisplatin-resistant tumors by activating the mTOR signaling pathway [293]. Kaempferol, another compound, exerts its anti-tumor effects by promoting autophagy through the restriction of the Met pathway [294]. Furthermore, GMI from Ganoderma microsporum has been shown to decrease viability in pemetrexed-resistant lung cancer cells by inducing autophagy [171].

### Challenges in clinical translation

Despite the promising results of autophagy modulation in preclinical models and early-phase clinical studies, substantial challenges remain in translating these strategies into safe and effective anticancer therapies.

First, the role of autophagy in tumors is highly context-dependent: it can function as a tumor-suppressive mechanism by maintaining genomic stability and clearing cellular stress, yet in established cancers it may evolve into an adaptive survival pathway that helps cells cope with therapeutic pressure. Most clinical trials to date have not stratified patients according to autophagy dependence, genetic background, or the activation status of autophagy pathways, and dynamic, systematic pharmacodynamic biomarkers to guide dosing and treatment duration are largely lacking.

Second, the autophagy inhibitors currently in clinical use are predominantly repurposed chloroquine derivatives, which suffer from limited selectivity. How to achieve effective inhibition of the lysosome/autophagy pathway while keeping off-target effects within an acceptable range remains unresolved. With the ongoing development and optimization of small-molecule inhibitors targeting VPS34, ULK1, PPT1 and selective autophagy receptors, a new generation of more selective agents may partially overcome the limitations of traditional chloroquine-based drugs. In parallel, AI-assisted drug discovery-integrating structure-based modelling, virtual screening and activity prediction-offers an opportunity to systematically identify and optimize autophagy-targeted compounds with improved specificity and drug-like properties [295].

Third, autophagy modulation as monotherapy rarely yields robust antitumor efficacy, which may partly explain the repeated failures of prior clinical efforts. At present, an increasing number of studies are evaluating autophagy-targeted approaches in combination with chemotherapy and targeted therapies to overcome current bottlenecks. In parallel, given the role of autophagy in supporting immune evasion and sustaining an immunosuppressive tumor microenvironment, combining autophagy modulation with immunotherapy has emerged as another promising avenue worthy of deeper investigation. Beyond conventional combination strategies, leveraging synthetic lethality as a therapeutic design framework is also gaining traction. In PDAC, dual inhibition of PIKfyve and the KRAS-MAPK axis can generate a metabolism-centered synthetic lethal interaction and markedly eliminate tumor burden across multiple preclinical human and mouse models. Mechanistically, PIKfyve is a lipid kinase essential for maintaining lysosomal membrane lipid homeostasis and lysosomal function; its inhibition compromises lysosomal activity and triggers metabolic rewiring, forcing tumor cells to rely on a KRAS-MAPK-driven compensatory lipogenic program. Subsequent blockade of KRAS-MAPK disables this critical bypass, thereby converting adaptive metabolic reprogramming into a lethal vulnerability [296].

## Summary and future perspectives

As research on autophagy in tumor initiation, progression, and therapeutic resistance continues to deepen, the prospects for autophagy-targeted therapy remain promising. Looking ahead, however, substantial work is still required to bridge fundamental autophagy biology and its successful translation into clinical practice.

Technologies for dynamically monitoring autophagic activity in vivo represent an important research direction. However, commonly used autophagy readouts, such as LC3 lipidation, p62/SQSTM1 degradation, and tandem fluorescent LC3 reporter systems, are still largely restricted to in vitro assays or ex vivo tissue analyses, which is clearly not conducive to real-time assessment of autophagy at the whole-organism level. In contrast, different classes of small-molecule fluorescent probes can selectively visualize endogenous lysosomal enzyme activities or the degree of lysosomal acidification, enabling dynamic monitoring of lysosomal functional changes in vivo and thereby allowing, to some extent, spatiotemporal tracking of the autophagic process [297]. Liquid biopsy systems integrating circulating proteins, autophagy-modulating miRNAs/circRNAs and exosomes may enable longitudinal, time-resolved monitoring of tumor autophagy dynamics [298]. Notably, some studies have reported that the therapeutic efficacy of HCQ is associated with smoking status, possibly mediated by elevated carbon monoxide levels. This finding suggests that certain metabolic or exposure-related features may also serve as indirect indicators of autophagic activity [299]. In the future, by integrating molecular imaging probes and liquid biopsy biomarkers with such clinical and metabolic characteristics, it may be possible to establish a multidimensional, time-resolved autophagy monitoring system, thereby providing a more precise basis for optimizing the timing of autophagy-targeted interventions and evaluating their therapeutic efficacy.

In recent years, nanomaterials have gradually emerged as powerful tools for modulating autophagy in the context of cancer therapy. Owing to their unique physicochemical properties and highly customizable functional design, nanocarriers can be precisely engineered to deliver autophagy modulators directly to tumor sites, thereby enhancing therapeutic selectivity while minimizing off-target toxicity. Various types of polymers, such as collagen, gelatin, PLGA, and albumin, can be used to construct diverse polymeric nanoparticles, which in turn can achieve the co-delivery of anticancer agents in combination regimens [300, 301]. With the clinical success of several siRNA-based drugs, including inclisiran, autophagy-targeted interventions built upon next-generation delivery platforms are poised to become a key breakthrough direction for the translational application of autophagy-targeted therapy. By using finely engineered delivery systems to transport shRNA/siRNA against ATGs into tumor cells, it is possible not only to implement stratified and precise intervention at distinct stages of the autophagy pathway, but also, to some extent, to overcome the constraints of traditionally undruggable targets and mitigate the specificity issues arising from the polypharmacology of existing small-molecule agents [302, 303].

In conclusion, autophagy plays a complex and multifaceted role throughout the cancer process, yet its development as a therapeutic target remains a long-term challenge. Systematically elucidating the dual characteristics of autophagy in a context-dependent manner, improving the precise measurement of autophagic flux, and developing more selective autophagy inhibitors or activators will collectively drive the clinical translation of autophagy-targeted strategies.

## Data Availability

Not applicable.
